# Analysis of gut microbiome composition, function, and phenotype in patients with osteoarthritis

**DOI:** 10.3389/fmicb.2022.980591

**Published:** 2022-11-25

**Authors:** Su Liu, Guoqing Li, Yuanchao Zhu, Chang Xu, Qi Yang, Ao Xiong, Jian Weng, Fei Yu, Hui Zeng

**Affiliations:** ^1^Department of Bone and Joint Surgery, Peking University Shenzhen Hospital, Shenzhen, China; ^2^National and Local Joint Engineering Research Center of Orthopaedic Biomaterials, Peking University Shenzhen Hospital, Shenzhen, China; ^3^Peking University Shenzhen Hospital Intelligent Hospital Research Academy, Shenzhen, China; ^4^Department of Ultrasonography, Peking University Shenzhen Hospital, Shenzhen, China

**Keywords:** gut microbiome, inflammation, osteoarthritis, stool, 16S rRNA sequencing

## Abstract

Gut microbiome (GMB) disturbance can induce chronic low-grade inflammation, which is closely related to the occurrence and development of osteoarthritis (OA). However, the relationship between GMB and OA remains unclear. In this study, we collected stool samples from OA patients and healthy people, and performed Alpha diversity, Beta diversity, MetaStat, and LEfSe analysis by 16S rRNA sequencing to find out the species with significant difference between the two groups. Random forest analysis was performed to find out biomarkers that could distinguish between OA patients and healthy people. PICRUSt and Bugbase analysis were used to compare the difference in functions and phenotypes. Multivariate linear regression analysis (MaAsLin) was used to adjust for gender, age, and body mass index (BMI). The results showed that there was a significant difference in the overall composition of GMB between the two groups (*p* = 0.005). After adjusting for gender, age, and BMI, we found that p_*Bacteroidota* (*Q* = 0.039), c_*Bacteroidia* (*Q* = 0.039), and o_*Bacteroidales* (*Q* = 0.040) were enriched in the OA group, while s_*Prevotella_copri* (*Q* = 0.001) was enriched in the healthy control group. *Prevotella* could distinguish between OA patients and healthy people with a better diagnostic power (AUC = 77.5%, *p* < 0.001, 95% CI: 66.9–88.1%). The functions of DNA transcription, amino acid metabolism (including histidine, lysine, and isoleucine), ATP metabolism, and phospholipid metabolism significantly decreased, while glucose metabolism, protein acetylation, and aspartate kinase activity significantly increased in the OA group. In terms of phenotypes, we found that the relative abundance of aerobic (*p* = 0.003) and Gram-negative (*p* < 0.001) was higher in the OA group, while contains mobile elements (*p* = 0.001) and Gram-positive (*p* < 0.001) were higher in the healthy control group. Our study preliminarily demonstrated that there were differences in the composition, function, and phenotype of GMB in stool samples between OA patients and healthy people, which provided a novel perspective on further study in OA.

## Introduction

Osteoarthritis (OA) is a chronic degenerative disease characterized by the degeneration and loss of articular cartilage, in which the destruction of extracellular matrix plays a significant role (Jin et al., 2022). It is reported that an estimated 7% of the population around the world has affected by OA, leading to a heavy burden on individuals and the social economy (David et al., 2020; Cook et al., 2022). The pathogenesis of OA is complex, among which mechanical factors and genetic predisposition are considered the predominant pathogenic factors (Johanne et al., 2016). OA has been identified as a non-inflammatory disease for a long time, but increasing evidence show that chronic low-grade inflammation seems to play a significant role in the occurrence and progression of OA, which is closely related to gut microbiome (GMB; Cindy et al., 2019). In 2019, the European Society for Economic Aspects of Osteoporosis, Osteoarthritis and Musculoskeletal Disease (ESCEO; Emmanuel et al., 2019) pointed out that the increased prevalence of OA is not only due to the extension of human life expectancy in modern society, but also on account of the alteration of modern lifestyle, especially the lack of physical exercise and low-fiber, high-sugar, and high-fat diet. This idea not only provides a better explanation for the high incidence of OA at present, but also proposes a novel perspective for the study of OA, that is the GMB.

GMB is a complicated ecosystem. The number of GMB in a healthy adult is about 10^13^–10^14^, which is equivalent to the cells in the entire human body (Pabst, 2017). GMB gradually increases after birth, reaching an adult state around age 2. It is influenced by a variety of factors, including diet, environment, genetics, drugs, immune system, gender, and age (Eckburg et al., 2005; Sekirov et al., 2010; Flint et al., 2012). Four phyla dominate the human GMB, of which *Firmicutes* and *Bacteroidetes* account for about 90%, followed by *Proteobacteria* and *Actinomycetes* with lower levels (Kim et al., 2017a). GMB plays a fundamental role in host homeostasis, with participating in host digestion by fermenting indigestible food residues, absorbing nutrients, hydrolyzing polysaccharides, synthesizing vitamins (K, B12, and B8), and absorbing fatty acids, and ultimately affecting host central nervous system and immune function (Weinstock, 2012). Do all diseases begin in the gut? The hypothesis was proposed by an ancient Greek physician Hippocrates nearly 2,500 years ago (Ramires et al., 2022). Continuous breakthroughs in modern medicine have changed our classical understanding of the gastrointestinal tract (GIT) and human health. Although the GIT has been shown to be a core component of human health under standard metabolic conditions, GMB composition and function have also been found to be closely linked to the development of a variety of disease, especially musculoskeletal disorders (Kundu et al., 2017; de Guchte et al., 2018; Lazar et al., 2018; Li et al., 2020). Yu et al. (2021) performed two-sample Mendelian randomization approach to reinforce the causal role of GMB in homeostasis, thereby contributing to the development and progression of OA. Researchers have primarily established mouse models of OA through high-fat diet, mechanical overloading, surgical induction, and gene editing techniques, and in all cases, GMB seemly contributed to OA progression (Favazzo et al., 2020). In the background of human biology, studies have shown that patients with OA exhibited dramatical dysbiosis of GMB, indicating pathogenic OA associated microbial shift (Yusuf et al., 2010; Griffin et al., 2012; Favazzo et al., 2020). This reinforced the notion that GMB may be out of balance under biological stress, thereby promoting pathological changes and leading to manifestations of various diseases, especially OA.

GMB maintains a balanced state in the host under physiological condition, playing an important role in various functions, such as promoting nutrient and drug metabolism, maintaining the integrity of gastrointestinal mucosal barrier, regulating immunity, and defending against exogenous pathogens, etc. (Sai et al., 2015). Disturbance of GMB can lead to metabolic disorders and inflammatory state in the body, both of which are critical factors for the progression of OA (Michelle and Wendy, 2016). The GMB produces a wide range of biological active molecules, such as short-chain fatty acid, protein, and enzymes, some of which are secreted in vesicles. These vesicles are insensitive to proteases, and can be distributed to other tissues or organs through blood circulation. At the same time, some of these products have an influence on the permeability of gastrointestinal mucosa, such as short chain fatty acid can trigger the permeability increased, leading to the Gram-negative bacteria ingredients (such as lipopolysaccharide (LPS)) take into the blood circulation, inducing inflammatory response by activating toll-like receptors (Umeharu et al., 2012; Chia et al., 2015; Maria and Richard, 2015). In addition, Wu et al. (2018) found that the levels of LPS and lipopolysaccharide-binding protein (LBP) in mice under sterile conditions were dramatically lower than those in the normal group, and were significantly associated with reduced osteophyte development. Meanwhile, Collins et al. (2015) reported that the abundance of *Lactobacillus* and *Methanobrevibacter* in animal feces of the experimental group with high sugar and fat was strongly associated with the modified Mankin score, and the ratio of *Firmicutes* was significantly higher than that of the control group. All of these studies mentioned above suggest that there may be a potential association between OA and GMB. However, the studies involving OA and GMB are extremely limited up to now.

The involvement of GMB in inflammation and degenerative musculoskeletal diseases is under continuous investigation, but no consensus has been reached. For this reason, we collected the stool samples from patients with OA and healthy controls, in order to find out the difference in composition, function, and phenotype of GMB between the two populations by using 16S rRNA sequencing. Further, we expect to find out the genera and biomarkers specific to OA, so as to provide a novel perspective on further study in OA.

## Materials and methods

### Study design and sample collection

Patients admitted to Peking University Shenzhen Hospital for knee OA from April 2021 to August 2021 were included as the OA group, and the subjects were selected from the healthy population as the healthy control group by 1: 1 sex matching. All patients and healthy controls signed written informed consent. All patients with primary knee OA were strictly diagnosed according to the American College of Rheumatology Guidelines (Balint and Szebenyi, 1996; Marc et al., 2012). All healthy controls were aged from 20 to 30 years and had no history of any knee pathologies or recent injuries. All subjects were Han Chinese from similar geographic areas (Shenzhen, a city in southern China) with similar eating habits, and were excluded for these reasons: (1) Suffering or previous suffered from any other osteoarticular diseases (such as rheumatoid arthritis, traumatic arthritis, inflammatory arthritis, etc), surgery, or joint cavity injection; (2) Taking any drugs that may affect the growth, composition, or distribution of GMB within 1 month of sampling, such as antibiotics, probiotics or prebiotics, corticosteroids, and other hormones; (3) Special diet (yogurt, alcohol, etc.) within 7 days of sampling; (4) Had a history of gastrointestinal diseases (such as irritable bowel syndrome, inflammatory bowel disease, tumor, et al.) and gastrointestinal surgery; (5) Had comorbidities of metabolic diseases (such as diabetes, hyperthyroidism, and hyperlipidemia, etc.), infectious diseases, immune system diseases or systemic diseases (such as cardiac insufficiency, renal insufficiency, etc.). Basic clinical characteristics including gender, age, body mass index (BMI), and Kellgren–Lawrence (K–L) grade were recorded (Kellgren and Lawrence, 1957). Qualified stool sample collected from each subject was about 3 g, immediately frozen in liquid nitrogen for 15 min, and then stored in-80°C freezer for further analysis.

### DNA extraction and sequencing

The total genome DNA was extracted by Magnetic Soil and Stool DNA Kit (TianGen, Beijing, China). DNA concentration and purity were monitored on 1% agarose gels. According to the concentration, DNA was diluted to 1 ng/μl with sterile water. 16S rRNA genes in V3–V4 were amplified with the primer 341F (5′-CCTAYGGGRBGCASCAG-3) and 806R (5′-GGACTACNNGGGTATCTAAT-3′). All PCR mixture contained 15 μl of Phusion^®^ High-Fidelity PCR Master Mix (New England Biolabs), 0.2 μM of each primer, and 10 ng target DNA, and cycling conditions consisted of a first denaturation step at 98°C for 1 min, followed by 30 cycles at 98°C (10s), 50°C (30s), and 72°C (30s) and a final 5 min extension at 72°C. Mix an equal volume of 1 × loading buffer (contained SYB green) with PCR products and perform electrophoresis on 2% agarose gel for DNA detection. The PCR products were mixed in equal proportions, and then Qiagen Gel Extraction Kit (Qiagen, Germany) was used to purify the mixed PCR products. Following the manufacture’s recommendations, sequencing libraries were generated with NEBNext® Ultra™ IIDNA Library Prep Kit (Cat No. E7645). The library quality was evaluated on the Qubit^®^ 2.0 Fluorometer (Thermo Scientific) and Agilent Bioanalyzer 2,100 system. Finally, the library was sequenced on an Illumina NovaSeq platform and 250 bp paired-end reads were generated.

### Paired-end reads merged and quality control

Paired-end reads were assigned to samples based on their unique barcodes and were truncated by cutting off the barcodes and primer sequences. Paired-end reads were merged using FLASH (Version 1.2.11; Magoc and Steven, 2011)^1^, a very fast and accurate analysis tool designed to merge paired-end reads when at least some of the reads overlap with the reads generated from the opposite end of the same DNA fragment, and the splicing sequences were called Raw Tags. Quality filtering on the raw tags was performed using the fastp (Version 0.20.0) software to obtain high-quality Clean Tags. The Clean Tags were compared with the reference database using Vsearch (Version 2.15.0) to detect the chimera sequences, and then, the chimera sequences were removed to obtain the Effective Tags (Brian et al., 2011).

### Amplicon sequence variants (ASVs) denoise and species annotation

For the Effective Tags obtained previous, denoise was performed with DADA2 module in the QIIME2 software (Version QIIME2-202006) to obtain initial ASVs, and then ASVs with abundance less than 5 were filtered out (Li et al., 2020). Species annotation was performed using Mothur with the database Silva 138.1. In order to study phylogenetic relationship of each ASV and the difference of the dominant species among different samples, multiple sequence alignment was performed using QIIME2 software. The absolute abundance of ASVs was normalized using a standard of sequence number corresponding to the sample with the least sequences. Subsequent analysis of alpha diversity and beta diversity were all performed based on the output normalized data.

### Alpha diversity analysis

In order to analyze the diversity, richness, and uniformity of the communities in the sample, Alpha diversity was calculated from three indices in QIIME2, including Observed_otus, Shannon, and Good’s_coverage.

### Beta diversity analysis

In order to evaluate the complexity of the community composition and compare the differences between groups, Beta diversity was calculated based on weighted and unweighted UniFrac distances in QIIME2. Principal Coordinate Analysis (PCoA) was performed to obtain principal coordinates and visualized differences of samples in complex multi-dimensional data. A matrix of weighted and unweighted UniFrac distances among samples obtained previously was transformed into a new set of orthogonal axes, where the maximum variation factor was demonstrated by the first principal coordinate, and the second maximum variation factor was demonstrated by the second principal coordinate, and so on. The three-dimensional PCoA results were displayed using QIIME2 package, while the two-dimensional PCoA results were displayed using ade4-package and ggplot2 package in R software (Version 2.15.3). To study the significant difference in community structure between groups, the Anosim analysis in the QIIME2 software was performed. To find out the significantly different species at each taxonomic level (Phylum, Class, Order, Family, Genus, Species), the R software (Version 3.5.3) was used to do MetaStat analysis. The linear discriminant analysis (LDA) effect size (LEfSe) software (Version 1.0) was used to do LEfSe analysis (LDA score threshold: 4) so as to find out the biomarkers. Random forest model was constructed by selecting different numbers of species at each level according to the gradient (Breiman, 2001). Significant species were screened out by Mean Decrease Accuracy and Mean Decrease Gin, and then receiver operating characteristic (ROC) curve was used to evaluate the performance of the model. Further, to study the functions of community species between groups, the Phylogenetic Investigation of Communities by Reconstruction of Unobserved States 2 (PICRUSt2) software (Version 2.1.2-b) was used to impute microbiome metagenomes from 16S rRNA sequencing, and function annotation was applied using the Clusters of Orthologous Groups of Proteins (COGs), enzyme nomenclature (EC), Kyoto Encyclopedia of Genes and Genomes (KEGG), KEGG orthology functional orthologs (KO), Protein Families Database (PFAM), and Institute for Genomic Research’s database of Protein Families (TIGRFAM) databases. Finally, the phenotypes present in each sample were predicted by BugBase from nine aspects: aerobic, anaerobic, contains mobile elements, facultatively anaerobic, forms biofilms, Gram negative, Gram positive, potentially pathogenic, and stress tolerant.

### Statistical analysis

All data were analyzed with SPSS (IBM version 25.0, Chicago, IL, United States) and R (Version 3.5.3, Vienna, Austria). For continuous variables, Student’s *t-*test was applied for normal distribution, while Mann–Whitney *U*-test for non-normal distribution. Chi-square test and Fisher’s exact test were applied to test categorical variables. Kruskal–Wallis rank sum test was used to analyze species with significant differences between the OA group and the control group. To gain more insight into which microbiome and function drive the association with knee OA, we performed multivariate linear regression analysis (MaAsLin) adjusting for gender, age and BMI. *p* value or corrected *p* value (*Q* value) < 0.05 was considered as statistical difference.

## Results

### Basic characteristics of two groups

There were 80 subjects included in the study, including 40 patients in the OA group and 40 subjects in the healthy control group. Each group consisted of 30 women (75%) and 10 men (25%), and there was no statistical difference in gender between the two groups. The median age of the OA group was 68 years (53–80 years), and that of the control group was 26 years (24–28 years), indicating a significant difference in age between the two groups (*p* < 0.001). The median BMI of the OA group was 25.71 kg/m^2^ (20.40–40.00 kg/m^2^), and that of the control group was 20.20 kg/m^2^ (17.85–23.94 kg/m^2^), indicating a significant difference in BMI between the two groups (*p* < 0.001). There were 16 cases (40%) with K–L grade III and 24 cases (60%) with K–L grade IV (Supplementary Figure S1). The basic characteristics of the two groups are shown in Table 1.

**Table 1:** Basic characteristics of the OA and control group.

Variables	Categories	OA (*N* = 40)		Control (*N* = 40)		*p*
		Count	Description	Count	Description	
Gender	Female	30	75%	30	75%	1
	Male	10	25%	10	25%	
Age (years)	Median	68		26		<0.001^**^
BMI (kg/m^2^)	Median	25.71		20.20		<0.001^**^
K-L grade	III	16	40%	—	—	—
	IV	24	60%	—	—	

OA, osteoarthritis; N, number; BMI, body mass index; K-L, Kellgren-Lawrence.

** *p* < 0.001, dramatically statistical difference.

### General information of sample sequencing

Eighty stool samples were sequenced, and the detailed sequencing data of each sample are shown in Supplementary Table S1. In the microbiome study, the median reads of original obtained were 110,676 (range from 76,363 to 130,925), and that of high qualified for final analysis was 74,987 (range from 44,893 to 98,207), with a median length of 416 nt (range from: 409 to 423 nt), and a median effective rate of 67.66% (range from 57.56 to 84.37%).

### Amplicon sequence variant analysis

Each sequence after dereplication by DADA2 was called ASV, in which the similarity of sequences was close to 100%. Based on the results of annotation and the characteristics of each sample, we obtained the species abundance at six levels: phylum, class, order, family, genus, and species. In this study, a total of 14,218 ASVs were obtained from 80 samples, of which 10,573 ASVs were obtained from the OA group, and 6,801 ASVs were obtained from the control group. There were 7,417 ASVs unique to OA group, and 3,645 ASVs unique to control group. A total of 3,156 ASVs were shared by the two groups.

The top 10 species with the highest abundance of each sample and each group were selected at six levels (phylum, class, order, family, genus, and species) according to the results of annotation. The histograms of relative abundance were generated to visually observe the species with high relative abundance at various taxonomic levels. All of the histograms of relative abundance between groups and samples were shown in Supplementary Figures S2, S3. From the results, we can find out that the dominant species were *Firmicutes*, *Bacteroidota*, *Proteobacteria*, and *Actinobacteriota* in both two groups at phylum level, accounting for 90.9% in the OA group and 96.6% in the control group. At genus level, microbiome with low abundance accounted for a larger proportion in the OA group (42.6%) and control group (39.2%). Among the top 10 genera in relative abundance, *Escherichia-Shigella*, *Bacteroides*, and *Faecalibacterium* were dominant in the OA group, accounting for 11.4, 15.7, and 12.0%, respectively. However, in the control group, *Escherichia-Shigella*, *Prevotella*, and *Faecalibacterium* were dominant, accounting for 9.7, 6.5, and 22.1%, respectively (Table 2).

**Table 2:** Relative abundance of top 10 species between groups at phylum and genus level.

Phylum			Genus		
	OA	Control		OA	Control
*Firmicutes*	40.7%	59.7%	*Escherichia-Shigella*	11.4%	9.7%
*Bacteroidota*	25.6%	12.5%	*Prevotella*	5.5%	6.5%
*Proteobacteria*	18.3%	14.9%	*Akkermansia*	3.5%	0.1%
*Verrucomicrobiota*	3.6%	0.1%	*Bacteroides*	15.7%	4.5%
*Actinobacteriota*	6.3%	9.5%	*Faecalibacterium*	12.0%	22.1%
*Fusobacteriota*	1.0%	0.0%	*Bifidobacterium*	3.0%	6.0%
*Acidobacteriota*	1.6%	1.4%	*Klebsiella*	2.4%	1.8%
*Gemmatimonadota*	0.7%	0.8%	*Dialister*	2.3%	6.0%
*Desulfobacterota*	0.7%	0.1%	*Megasphaera*	0.2%	3.8%
*Myxococcota*	0.3%	0.3%	*Ruminococcus_gnavus_group*	1.3%	0.2%
*Others*	1.1%	0.7%	*Others*	42.6%	39.2%
Total	100%	100%	Total	100%	100%

OA, osteoarthritis.

### Alpha diversity analysis

**Figure 1:**
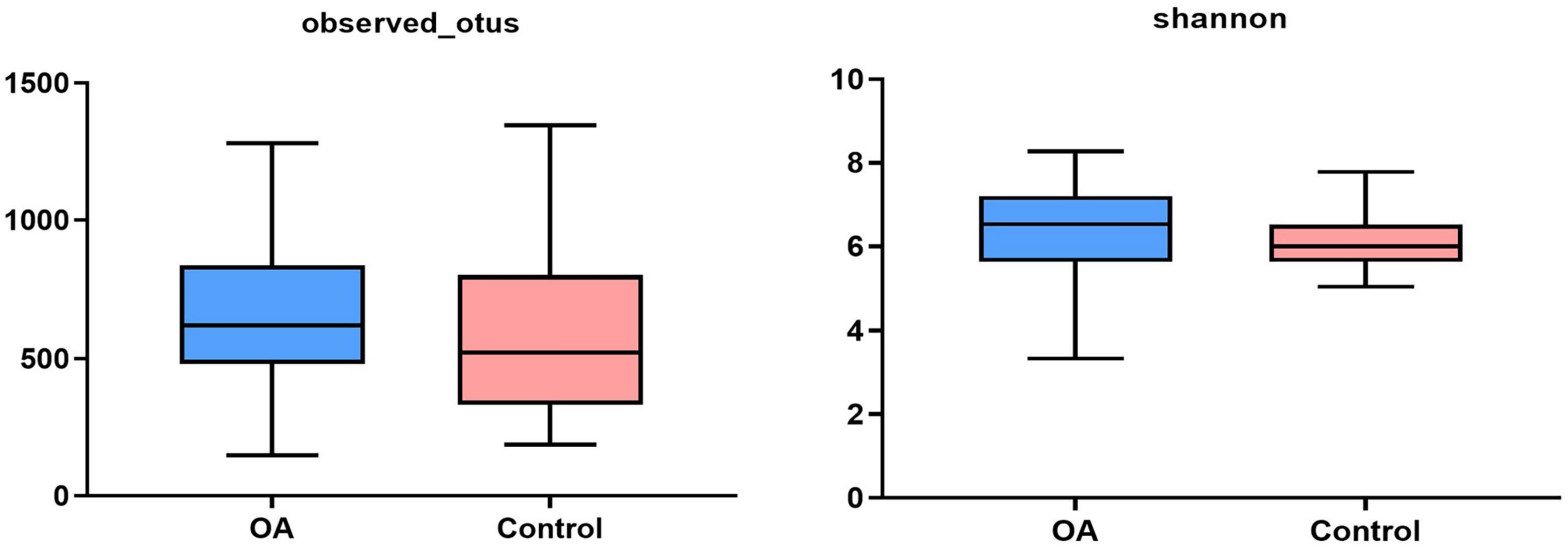
Alpha diversity indices between the two groups. OA, osteoarthritis.

Rarefaction curves were constructed (cut–off = 36,972) by randomly sampling from the collected sequences (Supplementary Figure S4). The results showed that the rarefaction curves of each index were tend to be flat, indicating basically reasonable depth of sample sequencing. The alpha diversity indices of all samples were analyzed and shown in Supplementary Table S2. From the results, we can find out that there was no statistical difference in all of the indices between the two groups (*p* > 0.05; Figure 1; Table 3). Meanwhile, we calculated the Goods’ Coverage of each sample, and the results showed that the sequencing coverage was more than 99%, indicating that the depth of sequencing had basically covered all species (Supplementary Table S2). Combined with the rarefaction curves and Goods’ Coverage, it was indicated that the sequencing in the study was reasonable, which could represent the biological community, and could be used for biodiversity analysis.

**Table 3:** Alpha diversity indices between the OA group and the control group.

Index	OA (median)	Control (median)	*H*	p^*^
Shannon	6.533	5.992	2.739	0.097
Observed_otus	621.500	523.000	2.723	0.099

OA, osteoarthritis.

* *p* < 0.05, statistical difference.

### Results of PCoA

**Figure 2:**
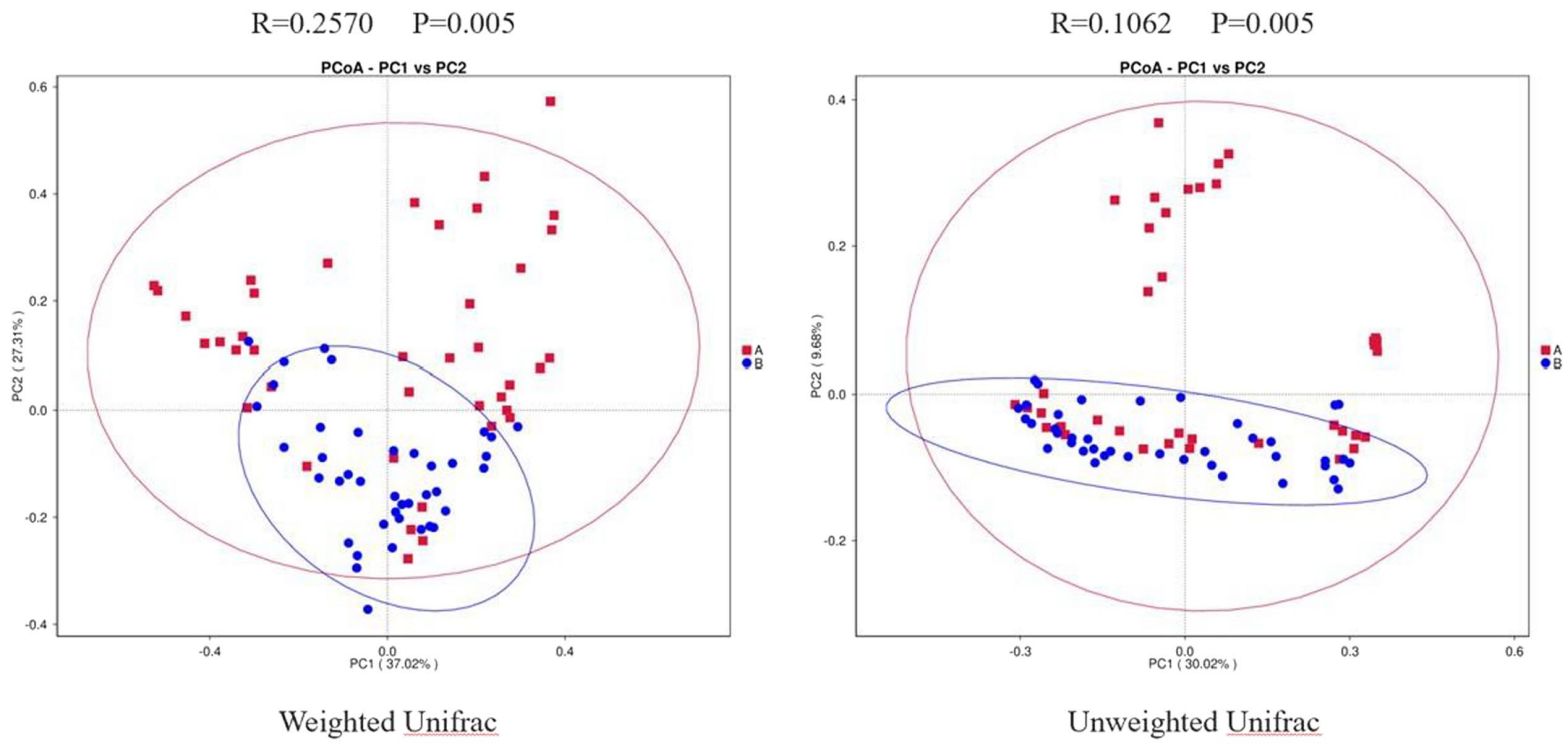
Two-dimensional PCoA Plots. The horizonal axis and vertical axis represent different principal components. Percentage represents the contribution of principal component to sample difference. A, osteoarthritis group; B, healthy control group.

Weighted Unifrac PCoA showed that the PC1 could explain 37.02% of the difference, and the PC2 could explain 27.31% of the difference. Unweighted Unifrac PCoA showed that the PC1 could explain 30.02% of the difference, and the PC2 could explain 9.68% of the difference. In addition, we performed Anosim analysis to further validate the difference between the two groups. The R value of weighted UniFrac PCoA was 0.2570, and that of unweighted UniFrac PCoA was 0.1062. Both weighted and unweighted UniFrac PCoA were statistically different between the two groups (*p* = 0.005; Figure 2). Combined with the results mentioned above, we speculated that there may be a dramatical difference in overall composition of GMB between the two groups.

### MetaStat analysis

**Figure 3:**
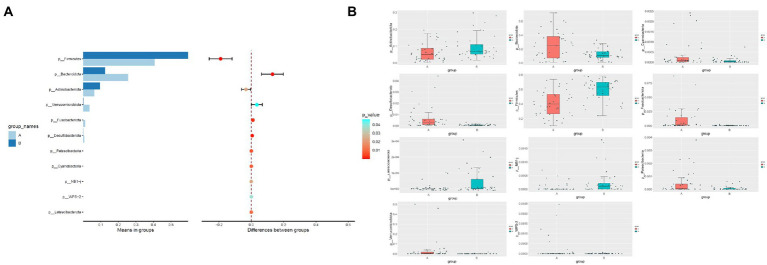
MetaStat Analysis. **(A)** Histogram of different species at phylum level. **(B)** Box plot of different species at phylum level. A, osteoarthritis group; B, healthy control group.

The results showed that there were 18 phyla with statistical difference at phylum level, mainly including *Firmicutes*, *Bacteroidota*, and *Actinobacteriota*. At class level, there were 39 classes with statistical difference, mainly including *Bacteroidia*, *Clostridia*, and *Actinobacteria*; at order level, there were 96 orders with statistical difference, mainly including *Bacteroidales*, *Oscillospirales*, and *Veillonellales-Selenomonadales*; at family level, there were 152 families with statistical difference, mainly including *Ruminococcaceae*, *Bacteroidaceae*, and *Veillonellaceae*; at genus level, there were 368 genera with statistical difference, mainly including *Bacteroides*, *Faecalibacterium*, and *Bifidobacterium*; at species level, there were 280 species with statistical difference, mainly including *Bacteroides_plebeius*, *Dialister*, and *Bacteroides_vulgatus* (Supplementary Tables S3–S8). The difference of dominant phyla between the two groups at phylum level is shown in Figure 3.

### LEfSe analysis

**Figure 4:**
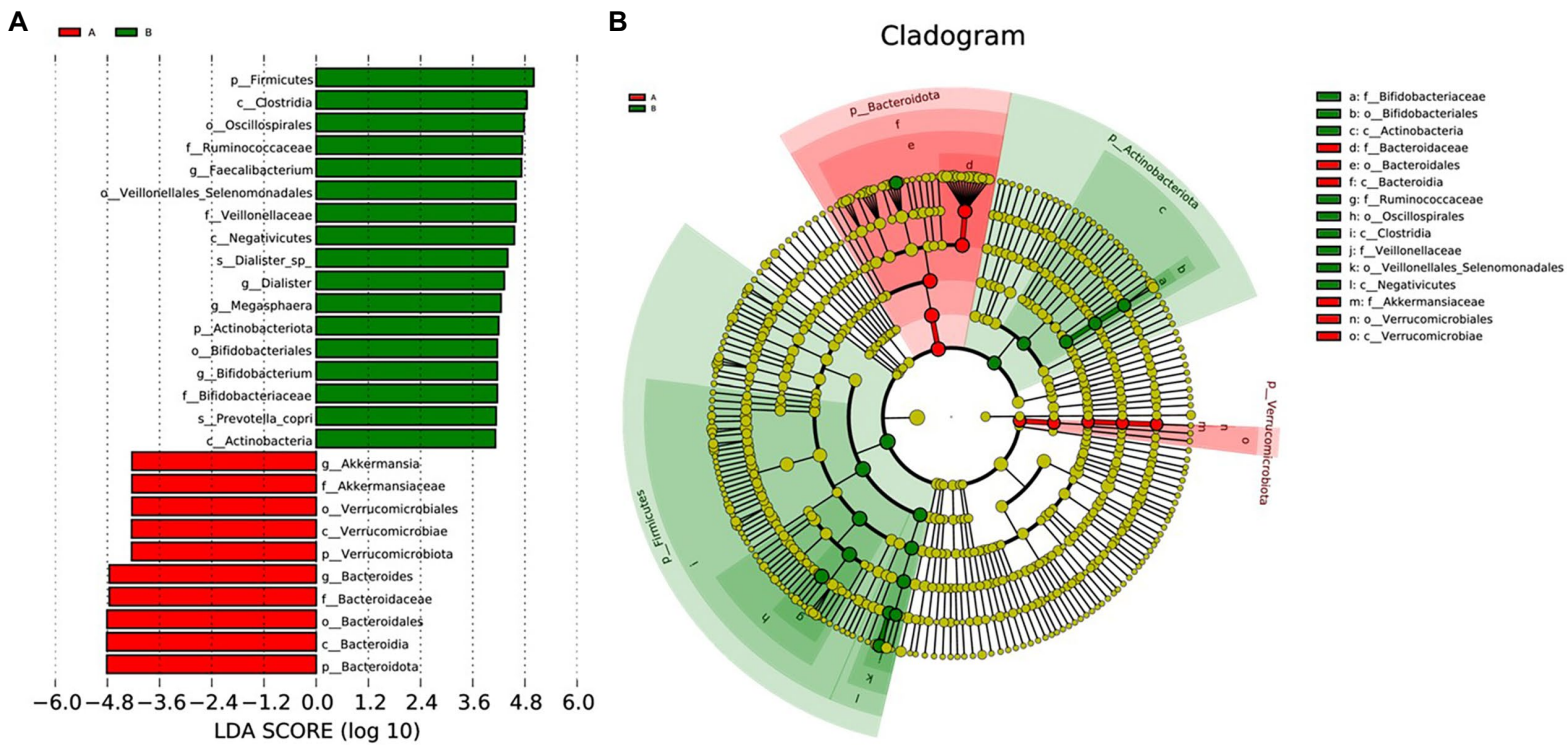
LEfSe analysis. **(A)**, Histogram of LDA Score distribution. The difference of species abundance between two groups. **(B)**, Cladogram. The phylogenetic distribution of GMB associated with two groups. The circle radiating from inside out represents the rank of classification from phylum to species.

We performed LEfSe to generate a histogram of LDA score and a cladogram to identify the specific microbiome involved with OA [23] (Figures 4A,B). From the cladogram, we can find out that *Firmicutes* and *Actinobacteriota* were significantly enriched in the control group (*p* < 0.05), while *Bacteroidota* and *Verrucomicrobiota* were enriched in the OA group (*p* < 0.05). LDA distribution diagram analysis (LDA score > 4) showed a clear difference in the microbiome characterized by higher *Firmicutes*, *Clostridia*, *Oscillospirales*, *Ruminococcaceae*, *Ruminococcaceae*, *Veillonellales_Selenomonadales*, *Faecalibacterium*, *Veillonellaceae*, *Negativicutes*, *Dialister_sp., Dialister*, *Megasphaera*, *Actinobacteriota*, *Bifidobacteriales*, *Bifidobacterium*, *Bifidobacteriaceae*, *Prevotella_copri*, and *Actinobacteria* in the control group (*p* < 0.05). However, *Akkermansia*, *Akkermansiaceae*, *Verrucomicrobiales*, *Verrucomicrobiae*, *Verrucomicrobiota*, *Bacteroides*, *Bacteroidaceae*, *Bacteroidales*, *Bacteroidia*, and *Bacteroidota* were more abundant in the OA group (*p* < 0.05). The absolute abundance of these species was shown in Table 4. After adjusting for gender, age, and BMI by MaAsLin, p_*Bacteroidota* (*Q* = 0.039), c_*Bacteroidia* (*Q* = 0.039), o_*Bacteroidales* (*Q* = 0.040), and s_*Prevotella_copri* (*Q* = 0.001) were significantly different between the two groups (Table 4).

**Table 4:** Absolute abundance of species with significant difference between groups.

Taxa	OA	Control	*T/U*	*p*	*Q*
p_*Firmicutes*	24,914 ± 1,161^#^	39,842 ± 10,348^#^	6.062	< 0.001^**^	0.443
c_*Clostridia*	20,113 ± 10,652^#^	30,625 ± 8,366^#^	4.909	< 0.001^**^	0.339
p_*Actinobacteriota*	3,217^##^	4,498^##^	475.000	0.002^*^	0.717
p_*Verrucomicrobiota*	189^##^	25^##^	304.500	< 0.001^**^	0.330
p_*Bacteroidota*	15,589^##^	7,397^##^	539.000	0.012^*^	**0.039**^&^
c_*Negativicutes*	1,577^##^	5,788^##^	283.000	< 0.001^**^	0.332
c_*Actinobacteria*	1,656^##^	3,063^##^	441.000	0.001^*^	0.818
c_*Verrucomicrobiae*	189^##^	25^##^	305.000	< 0.001^**^	0.330
c_*Bacteroidia*	15,582^##^	7,395^##^	539.000	0.012^*^	**0.039**^&^
o_*Oscillospirales*	9,379^##^	19,287^##^	295.000	< 0.001^**^	0.144
o_*Veillonellales*-Selenomonadales	1,072^##^	5,772^##^	226.000	< 0.001^**^	0.499
o_*Bifidobacteriales*	396^##^	2,514^##^	325.000	< 0.001^**^	0.968
o_*Verrucomicrobiales*	127^##^	0^##^	329.500	< 0.001^**^	0.332
o_*Bacteroidales*	15,393^##^	7,368^##^	538.500	0.012^*^	**0.040**^&^
f_*Ruminococcaceae*	7,580^##^	16,851^##^	295.000	< 0.001^**^	0.115
f_*Veillonellaceae*	822^##^	5,423^##^	250.000	< 0.001^**^	0.295
f_*Bifidobacteriaceae*	396^##^	2,514^##^	325.000	< 0.001^**^	0.968
f_*Akkermansiaceae*	127^##^	0^##^	329.500	< 0.001^**^	0.332
f_*Bacteroidaceae*	9,053^##^	2,835^##^	346.000	< 0.001^**^	0.360
g_*Faecalibacterium*	5,406^##^	14,633^##^	281.000	< 0.001^**^	0.105
g_*Dialister*	691^##^	3,005^##^	386.000	< 0.001^**^	0.493
g_*Megasphaera*	52^##^	1946^##^	57.000	< 0.001^**^	0.230
g_*Bifidobacterium*	396^##^	2,514^##^	325.000	< 0.001^**^	0.968
g_*Akkermansia*	127^##^	0^##^	328.500	< 0.001^**^	0.331
g_*Bacteroides*	9,053^##^	2,835^##^	346.000	< 0.001^**^	0.360
s_*Dialister_sp*.	401^##^	2,999^##^	261.000	< 0.001^**^	0.419
s_*Prevotella_copri*	222^##^	2,824^##^	359.500	< 0.001^**^	**0.001**^&^

OA, osteoarthritis. Q, p value after adjusting for gender, age, and body mass index (BMI).

# mean ± standard deviation.

## median.

* *p* < 0.05, statistical difference.

** *p* < 0.001, dramatically statistical difference; and *Q* < 0.05, statistical difference.

### Random forest analysis

**Figure 5:**
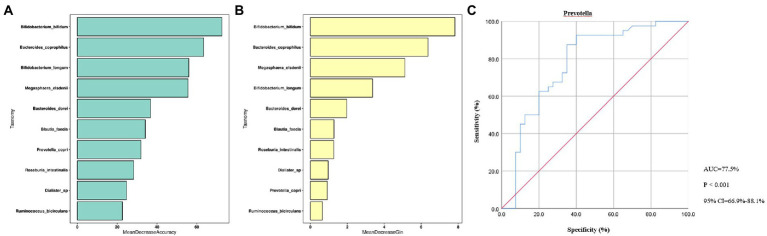
Random Forest Analysis. **(A)**, Mean Decrease Accuracy; **(B)**, Mean Decrease Gin; **(C)**, ROC curve. ROC, receiver operating characteristic; AUC, are under curve; CI, confidence interval; *p* < 0.01, dramatically statistical difference.

The random forest model was performed based on the species with differential abundance to detect potential diagnostic biomarkers that could be used to discriminate the OA and control groups. The optimal model provided the best discriminating power by using 10 species (Figures 5A,B). Combined with the above analysis, we finally screen out that *Prevotella* may be a potential diagnostic biomarker of the two groups. To explore the discriminating power of *Prevotella,* we performed ROC curves and calculated the area under the curve (AUC) values. As shown in Figure 5C, the AUC value of *Prevotella* was 77.5% (*p* < 0.001, 95% CI: 66.9–88.1%), indicating a better efficiency in discrimination.

### Predicted function

The results of PCA and Venn diagram are shown in Supplementary Figures S5, S6. The relative and absolute abundance of predicted functions (Top 30) in these databases were shown in Supplementary COG, KEGG, EC, KO, PFAM, and TIGRFAM. After adjusting for gender, age, and BMI, we can find out that the functions of DNA transcription, amino acid metabolism (including histidine, lysine, and isoleucine), adenosine triphosphate (ATP) metabolism, and phospholipid metabolism significantly decreased, while glucose metabolism, protein acetylation, and aspartate kinase activity significantly increased in the OA group. The detailed comparison of functions between groups is shown in Table 5.

**Table 5:** The absolute abundance of functions with statistical difference between groups.

Function	OA	Control	*p*	*Q*
COG0745	201,561^##^	221,739^##^	0.002^*^	0.028^&^
COG1309	159,156 ± 30,575^#^	178,693 ± 22,198^#^	0.002^*^	0.029^&^
COG0583	147,960^##^	190,206^##^	<0.001^**^	0.038^&^
COG2814	120,424 ± 30,219^#^	105,038 ± 30,318^#^	0.026^*^	0.025^&^
COG0451	113,247^##^	92,398^##^	<0.001^**^	0.034^&^
COG1670	94,183^##^	80,466^##^	<0.001^**^	0.006^&^
COG0834	62,511^##^	80,843^##^	0.008^*^	0.046^&^
COG2197	82,243^##^	59,843^##^	0.018^*^	0.020^&^
EC:2.7.13.3	156,797 ± 30,898^#^	170,494 ± 20,685^#^	0.022^*^	0.004
EC:3.6.3.14	77,826^##^	87,594^##^	0.032^*^	0.017^&^
EC:3.6.3.17	54,789^##^	77,270^##^	0.021^*^	0.034^&^
EC:3.5.1.28	74,091 ± 12,058^#^	62,421 ± 8,864^#^	<0.001^**^	0.002^&^
EC:2.2.1.1	54,671 ± 11,447^#^	59,216 ± 6,464^#^	0.032^*^	0.043^&^
K00615	54,670 ± 11,447^#^	59,216 ± 6,464^#^	0.032^*^	0.043^&^
K06889	57,086^##^	49,012^##^	0.027^*^	0.010^&^
K01448	53,281 ± 9,737^#^	46,816 ± 6,746^#^	0.001^*^	0.002^&^
K07025	53,809^##^	46,720^##^	<0.001^**^	0.019^&^
PWY-2942	54,670 ± 11,447^#^	59,216 ± 6,464^#^	0.032^*^	0.043^&^
PWY-5103	30,745^##^	30,916^##^	0.027^*^	0.010^&^
PWY-7220	53,281 ± 9,737^#^	46,816 ± 6,746^#^	0.001^*^	0.002^&^
PWY4FS-7	26,652^##^	30,609^##^	<0.001^**^	0.019^&^
PF00486	206,948^##^	224,016^##^	0.020^*^	0.015^&^
PF00534	201,035 ± 27,455^#^	188,176 ± 21,392^#^	0.022^*^	0.046^&^
PF00132	189,164^##^	181,314^##^	0.007^*^	0.003^&^
PF03466	151,293^##^	191,960^##^	<0.001^**^	0.034^&^
TIGR01484	95,285 ± 22,620^#^	113,310 ± 15,989^#^	<0.001^**^	0.042^&^
TIGR01726	63,149^##^	81,207^##^	0.008^*^	0.037^&^
TIGR01003	48,491 ± 17,615^#^	59,381 ± 10,966^#^	0.001^*^	0.042^&^
TIGR00517	51,602 ± 7,977^#^	55,620 ± 4,532^#^	0.007^*^	0.012^&^
TIGR00093	45,961 ± 8,023^#^	50,890 ± 5,906^#^	0.002^*^	0.025^&^
TIGR00813	42,018^##^	35,095^##^	0.013^*^	0.003^&^
TIGR00657	42,063 ± 5,555^#^	38,553 ± 4,467^#^	0.003^*^	0.012^&^

COG0745: DNA-binding response regulator, OmpR family, contains REC and winged-helix (wHTH) domain; COG1309: DNA-binding transcriptional regulator, AcrR family; COG0583: DNA-binding transcriptional regulator, LysR family; COG2814: Predicted arabinose efflux permease, MFS family; COG0451: Nucleoside-diphosphate-sugar epimerase; COG1670: Protein N-acetyltransferase, RimJ/RimL family; COG0834: ABC-type amino acid transport/signal transduction system, periplasmic component/domain; COG2197: DNA-binding response regulator, NarL/FixJ family, contains REC and HTH domains; EC:2.7.13.3: Histidine kinase; EC:3.6.3.14: H(+)-transporting two-sector ATPase; EC:3.6.3.17: Monosaccharide-transporting ATPase; EC:3.5.1.28: N-acetylmuramoyl-L-alanine amidase; EC:2.2.1.1: Transketolase; K00615: transketolase; K06889: uncharacterized protein; K01448: N-acetylmuramoyl-L-alanine amidase; K07025: putative hydrolase of the HAD superfamily; PWY-2942: L-lysine biosynthesis III; PWY-5103: L-isoleucine biosynthesis III; PWY-7220: adenosine deoxyribonucleotides de novo biosynthesis II; PWY4FS-7: phosphatidylglycerol biosynthesis I (plastidic); PF00486: Transcriptional regulatory protein, C terminal; PF00534: Glycosyl transferases group 1; PF00132: Bacterial transferase hexapeptide (six repeats); PF03466: LysR substrate binding domain; TIGR01484: HAD hydrolase, family IIB; TIGR01726: amino ABC transporter, permease protein, 3-TM region, His/Glu/Gln/Arg/opine family; TIGR01003: phosphocarrier, HPr family; TIGR00517: acyl carrier protein; TIGR00093: pseudouridine synthase; TIGR00813: transporter, solute:sodium symporter (SSS) family; TIGR00657: aspartate kinase. OA, osteoarthritis; Q, p value after adjusting for gender, age, and body mass index (BMI).

# mean±standard deviation.

## median.

* *p* < 0.05, statistical difference.

** *p* < 0.001, dramatically statistical difference; and & *Q* < 0.05, statistical difference.

### Predicted phenotype

**Figure 6:**
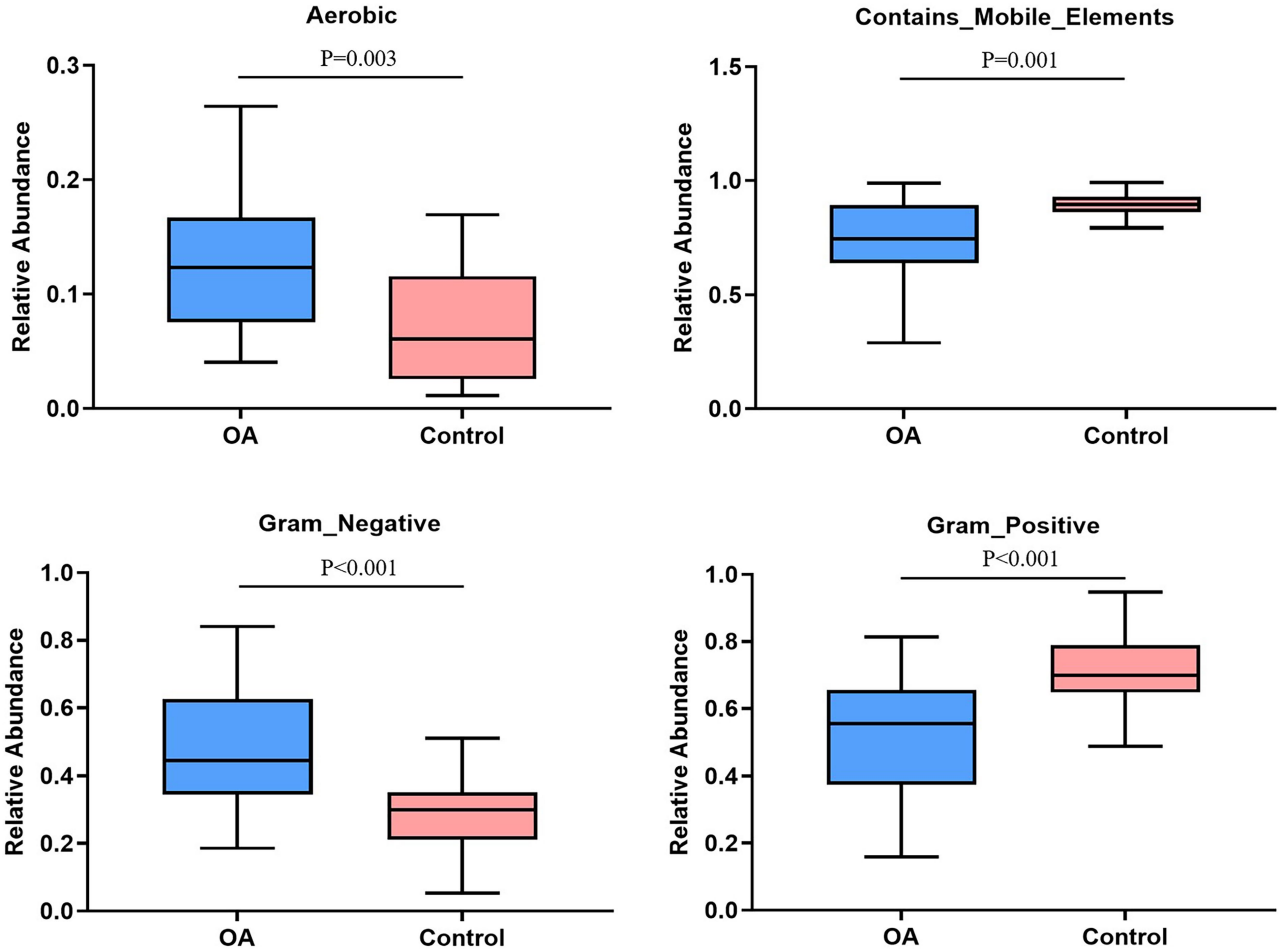
Phenotype analysis. The phenotypes with significant differences in relative abundance between groups. OA, osteoarthritis.

The relative abundance of aerobic was higher in the OA group (*p* = 0.003), mainly including *Acidobacteria*, *Actinobacteria*, *Proteobacteria*, and *Verrucomicrobia*, while *Acidobacteria*, *Actinobacteria*, and *Proteobacteria* were dominant in the control group. The relative abundance of Gram-negative was higher in the OA group (*p* < 0.001), dominated by *Acidobacteria*, *Bacteroidetes*, *Proteobacteria*, and *Verrucomicrobia*, while *Bacteroidetes*, *Proteobacteria*, and *Firmicutes* were dominant in the control group. In contrast, the relative abundance of Gram-positive was higher in the control group (*p* < 0.001), dominated by *Firmicutes* and *Actinobacteria*, while *Firmicutes* was dominant in the OA group. As for contains mobile elements, the relative abundance was higher in the control group (*p* = 0.001), with a major of *Firmicutes*, *Proteobacteria*, and *Actinobacteria*, while the mainly phenotypes in the OA group were *Firmicutes* and *Proteobacteria*. There was no significant difference in anaerobic, facultatively anaerobic, forms biofilms, potentially pathogenic, and stress tolerant between groups (Table 6; Figure 6).

**Table 6:** The relative abundance of phenotypes between groups.

Phenotypes	OA (median)	Control (median)	*U*	*p*
Aerobic	0.123	0.061	158	**0.003** ^*^
Anaerobic	0.722	0.798	247	0.204
Contains_Mobile_Elements	0.745	0.895	142	**0.001** ^*^
Facultatively_Anaerobic	0.072	0.099	253	0.248
Forms_Biofilms	0.263	0.238	292	0.691
Gram_Negative	0.445	0.300	111	**<0.001** ^**^
Gram_Positive	0.555	0.700	111	**<0.001** ^**^
Potentially_Pathogenic	0.606	0.565	215	0.059
Stress_Tolerant	0.085	0.089	288	0.635

OA, osteoarthritis;

* *p* < 0.05, statistical difference;

** *p* < 0.001, dramatically statistical difference.

The bold values indicate statistical differences.

## Discussion

OA is a chronic degenerative disease caused by multiple factors, and its pathogenesis has not been clarified clearly. In recent years, an increasing number of studies have reported that there may be a potential relationship between GMB and OA, that is, GMB may be involved in the occurrence and progression of OA through the “Gut-Joint” axis (Cindy et al., 2019; Emmanuel et al., 2019). However, due to the limited studies, the difference in GMB between OA patients and healthy people, as well as the impact of GMB on OA, have not been clarified. Therefore, we collected stool samples from OA patients and healthy people to explore the difference in composition, function, and phenotype of GMB by using 16S rRNA sequencing.

GMB is an ecological structure formed in the process of co-evolution between microbe and host, which plays an important role in individual growth and development (Michelle and Wendy, 2016). GMB can synthesize many compounds needed for its own survival and the survival of its host, providing a variety of enzymes and biochemical metabolic pathways for human metabolism (Michelle and Wendy, 2016). *Firmicutes* and *Bacteroidetes* are the two major phyla in GMB, which can fermentate dietary fiber to produce a variety of short-chain fatty acids (such as butyrate, propionate, and acetate), thus affecting host metabolism by acting on G protein-coupled receptors (GPCP) expressed by intestinal endocrine cells (Muhammad et al., 2021). Acetate and butyrate stimulate the release of glucagon-like peptide 1 (GLP-1) and peptide YY (PYY), while propionate enhances fat storage by inducing the secretion of growth hormone-releasing peptide (Charlotte et al., 2018). Succinic acid enhances thermogenesis of adipose tissue by driving the expression of uncoupling protein 1 (UCP 1). However, succinic acid has a pro-inflammatory effect on macrophages activated by lipopolysaccharide (LPS), which may contribute to adipose tissue inflammation and insulin resistance (Fan and Pedersen, 2021). GMB is involved in many complex metabolic processes in the host. The study simply found that there was a significant difference in several GMB functions between the two groups, and no further investigation was conducted to explore these specific functions. Whether these altered functions are related to the high incidence of OA and the underlying mechanism still needs to be clarified in the future.

GMB is a mediator of host nutrient metabolism, and changes in its composition will directly affect the host’s metabolism. At present, the research on the metabolism of OA patients mostly focused on urine, synovial fluid, blood, and articular cartilage. Yang et al. (2009) conducted a metabolomic analysis of the urine of knee OA patients and found that compared with the control group, the contents of aconitic acid, isocitrate, homovanillic acid, and histamine increased in the OA group. Anderson et al. (2018) conducted a metabolomics test on the synovial fluid of 10 OA patients. Compared with the healthy people, tyrosine, valine, alanine, 2-hydroxybutyric acid, inositol, acetylcholine, and adenosine significantly increased in the OA group. Meanwhile, Mickiewicz et al. (2015) reported that there were 11 metabolites quite different between OA patients and healthy people, including fructose, citrate, O-acetyl carnitine, N-phenylacetylglycine, methionine, ethanol, creatine, malate, ethanolamine, 3-hydroxybutyrate, and acetylcarnitine. A total of 114 metabolites were identified by metabolomic analysis of synovial fluid of OA patients with different degrees of lesions, and there were significant differences in the metabolic profiles between the early OA group and the late OA group. Substances such as malic acid, ethanolamine, squalene, glycerol, and myristic acid were the key metabolites that distinguish early from advanced OA, and these metabolites were related to fatty acid metabolism, glyceride metabolism, and the tricarboxylic acid cycle (Kim et al., 2017b). Yang et al. (2016) used UPLC/Q-TOF-MS technology to analyze the metabolism of articular cartilage and found that taurine metabolism and β-alanine metabolism may be related to subchondral bone sclerosis; taurine, 1-carnitine, and glycerophospholipid may play important regulatory roles in the pathological process of subchondral bone sclerosis; β-alanine and 1-carnitine may be associated with increased energy expenditure during sclerosis. In the blood metabolomics analysis Zhang et al. (2016) collected the plasma of OA patients and healthy people, and found that there were 6 biochemical substances related to OA, among which arginine played an important role in distinguishing the two populations. Arginine deficiency could activate the arginine-ornithine pathway and disrupt the balance between cartilage repair and damage. However, no metabolomic analysis based on human stool samples has been found to compare the metabolic function of GMB between OA patients and healthy people. In this study, 16S rRNA sequencing was used to preliminarily analyze the function of GMB in two populations. It can be seen from the results that the changes in the composition of GMB between the two groups led to significant differences in their functions, which were manifested in a significant decrease in DNA transcription, amino acid metabolism (histidine, lysine, and isoleucine), ATP metabolism and phospholipid metabolism in the OA group, while increased glucose metabolism, protein acetylation, and aspartokinase activity in the OA group. The results were at odds with the other tissue metabolomics described above. Metabolomic analysis of GMB in the feces of the two populations is required to find the link between GMB and OA at the metabolic level, as well as OA-specific metabolites.

Bone metabolism and musculoskeletal diseases are influenced by GMB and its metabolites. *Lachnospira* is a phylogenetically and morphologically heterogeneous taxon of the Firmicutes. It is an anaerobic bacterium that mainly inhabits the digestive tract of humans or animals. In recent years, studies have found that it is closely related to the bone density of the heel (Wei and Karsenty, 2018). *Ruminococcus* belongs to the Firmicutes. It mainly obtains energy by absorbing monosaccharides and degrading mucins, with acetic acid and formic acid as its fermentation metabolites. Researchers have found that it is closely related to inflammation and arteriosclerosis (Valenzano et al., 2020). At the same time Sun et al. (2020) reported that oral administration of probiotic metabolites Kefir-fermented peptides can prevent postmenopausal osteoporosis, and mildly modulate the structure of GMB in ovariectomy mice, resulting in an increase in the abundance of *Rumenobacteria*. *L. acidophilus*, as a genus of *Lactobacillus* in *Firmicutes*, often appears in our daily life. It mainly exists in the small intestine and releases lactic acid, acetic acid, and some antibiotics that act on harmful bacteria, but the bacteriostatic effect is relatively weak (Liao et al., 2022). The food containing *Lactobacillus acidophilus* is good for the digestive organs, for its capacity of restoring the normal balance of GMB and inhibiting the proliferation of spoilage bacteria. Lactic acid and acetic acid produced by *Lactobacillus acidophilus* can improve the utilization level of calcium and iron, promote the digestion of iron and vitamins, and reduce the absorption of cholesterol (Jiang et al., 2021). At the same time, studies have also confirmed that *Lactobacillus acidophilus* can act on the growth hormone axis and promote bone development in malnourished mice (Philbrick et al., 2015). Said and Kareem (2020) proved that oral administration of *Lactobacillus acidophilus* can down-regulate pro-inflammatory factors (IL-6 and TNF-α) and up-regulate anti-inflammatory (IL-10) cytokines, playing a role in bone protection for inflammation-related arthritis. Tariq et al. (2017) have found that *Lactobacillus acidophilus* exerts positive influence on bone in estrogen-deficient mice by regulating the ratio of TH17/Treg cells. *Clostridium butyricum* is a typical member of the *Bacillus*. *Clostridium butyricum*, or *butyric acid bacteria*, as a probiotic, exhibits many biological characteristics that are beneficial to the human body. Previous studies have shown that short-chain fatty acids (SCFAs), metabolites of *Clostridium butyricum*, such as propionate, acetate, and butyrate, exert positive effects on bone metabolism. SCFAs have been proved to be effective regulators of osteoclast metabolism and bone homeostasis *in vivo* and *in vitro*, and can be used as therapeutic targets to prevent bone loss caused by post-menopause and inflammation (Rosenblatt et al., 2016). Meanwhile, researchers further proposed the beneficial effect of *Clostridium butyricum* on bone development, confirming that oral administration of ID-CBT5101 (tyndallized butyricum) can alleviate OA symptoms by regulating the expression of inflammatory factors and bone metabolism (Li et al., 2020).

Recently, although research on the association between GMB and OA is increasing, no clear conclusions have been drawn. Exactly which genera play a role in the occurrence and development of OA remains unclear, and the results of related studies are inconsistent. The largest study to date was performed by Boer et al. (2019). They performed 16S sequencing in 1,427 knee OA patients and healthy individuals and replicated in an additional 867 patients with fecal samples. After adjusting for BMI, there was no significant difference in Alpha diversity between the two populations, which was consistent with the present study. In addition, they assessed pain perception in OA patients and measured joint effusion using magnetic resonance imaging, and found that both pain perception and joint effusion were significantly associated with *Streptococcus*, which was also validated in the replication cohort. Wei et al. (2021) used 16S sequencing to perform microbiological test on the stools of 72 symptomatic and 1,316 asymptomatic patients with hand OA. They also found no difference in Alpha diversity in GMB between the two groups, but noted an increase in *Bilophila* and *Desulfovibrio* and a decrease in *Roseburia*. At the same time, they observed that these genera were associated with amino acid, lipid, and carbohydrate metabolism pathways. Chen et al. (2021) performed whole-genome shotgun sequencing in 57 OA patients and 57 healthy controls, and found that Alpha diversity reduced in OA patients. They also identified 19 bacterial species, deceased tryptophan, glutamine, and tyrosine, as well as increased propionate closely related to OA. Microbiome analysis of 59 patients with combined hand and knee OA and 33 disease-free controls by Rushing et al. (2022) found that decreased *Clostridiales* was associated with increased incidence of OA. Wang et al. (2021) sequenced the fecal microbiota of 86 overweight knee OA patients and 96 overweight controls without OA, and identified 87 genera associated with OA. Yu et al. (2021) utilized large-scale genome-wide association study (GWAS) summary statistics to assess the causal relationship between GMB and OA. They performed a two-sample Mendelian randomization approach to identify three microbial taxa that were causally associated with OA: Methanobacteriaceae, Desulfovibrionales, and Ruminiclostridium 5 genus. A systematic review analyzing 37 original studies revealed that *Clostridium*, *Streptococcus*, *Bacteroides*, and *Firmicutes* were upregulated in OA subjects, while *Lactobacillus* and *Bifidobacterium* were associated with improved OA outcomes (Bonato et al., 2022). In rhesus monkeys with spontaneous OA, it was also found that compared with normal monkeys, the GMB of OA monkeys was significantly changed, manifested as an increase in the abundance of *Mollicutes*, *Tenericutes*, *Coprobacillus*, and *Faecalitalea* (Yan et al., 2021). In addition, in the context of OA, studies have reported the analysis of microbes in other sites outside the gut. Chen et al. (2018) assessed the oral microbiome of 155 healthy subjects, 110 rheumatoid arthritis patients, and 67 knee OA patients by 16S sequencing and found that Alpha diversity, as well as *Firmicutes*, *Streptococcus*, *Actinomyces*, *Ruminococcus*, and *Bifidobacterium* significantly increased in OA patients.

The composition and content of GMB varied greatly among different individuals, which were affected by various factors, such as age, region, diet, and physiological state (Daniel et al, 2007). After analysis, we found that there were significant differences in the overall composition of GMB between the two groups, mainly reflected in the absolute abundance of *Prevotella* in the control group was higher than that in the OA group. Combined with the random forest analysis, we concluded that *Prevotella* had a better diagnostic power in distinguishing between the OA and healthy control group, suggesting that *Prevotella* may be a potential diagnostic biomarker. *Prevotella* is a Gram-negative anaerobic bacteria belonging to *Bacteroidetes*, considered to function as a “probiotic” in humans. The result as consistent with Yan et al. (2021), who found that the abundance of *Prevotella* in rhesus monkeys with spontaneous OA was lower compared with the healthy. Meanwhile, researchers (Eric et al., 2016; Marietta et al., 2016; Baskar et al., 2019) isolated *Prevotella* from human gut and fed it to DQ8 mice, finding that the incidence and severity of arthritis significantly decreased compared with the control group. However, Olivia et al. (2021) reported that *Prevotella* was significantly enriched in obese women, and BMI was positively correlated with *Prevotella* abundance, suggesting that there may be a potential association between *Prevotella* and obesity. As we all know, obesity plays a significant role in the development of OA. The inconsistent results listed above indicated a complex mechanism between *Prevotella* and OA, leading to a debate about whether *Prevotella* is beneficial or harmful to human health (Adrian et al., 2021). It is now widely accepted that the diversity of *Prevotella* is positively correlated with intestinal homeostasis (Amira and Dirk, 2019; Carmen and Corrie, 2019). *Prevotella* has a potential ability to regulate the GMB, so a clear understanding of it in human metabolism and nutrition could contribute to clinical practice.

GMB is a complex microecosystem whose effects on the human body are affected by many factors, including the inner and outer environment of host (Christopher and Tiffany, 2018). We first predicted the phenotypes of GMB between the two groups, and found that the relative abundance of aerobic and Gram negative was higher in the OA group, while contains mobile elements, facultatively anaerobic, and Gram positive were higher in the control group. The above results were only a simple phenotype prediction of GMB, which only indicated that GMB may be disordered when OA occurs, and cannot draw a definite conclusion about the pathogenicity. Recently, studies have reported that GMB can produce a wide range of biological molecules, which have an effect on the permeability of gastrointestinal mucosa, allowing the metabolites to transport from the intestine to the blood circulation and then to the other tissues or organs in distance, including the LPS (Umeharu et al., 2012; Maria and Richard, 2015; Sai et al., 2015; Michelle and Wendy, 2016). After entering the systemic circulation from the “leaky intestine,” LPS can induce chronic low-grade inflammation in the host, indicating that the increased abundance of Gram-negative bacteria could participate in the pathological process of OA by enhancing the level of LPS (Mairi et al., 2015; Michelle and Wendy, 2016). Huang et al. (2016) found that elevated levels of LPS and lipopolysaccharide-binding protein (LBP) were associated with the severity of knee osteophytes and the abundance of activated macrophages in synovium. Exercise can alleviate inflammatory response induced by LPS through regulating GMB composition, thus contributing to the treatment of OA (Li et al., 2021). In addition, as mentioned above, the aspartate kinase activity significantly increased in the OA group. As a study reported, the aspartate kinase is produced by *Escherichia coli* (Jens and Volker, 2011). Therefore, we speculated that the occurrence and progression of OA may be closely related to the increased abundance of Gram-negative bacteria. However, we only made a preliminary elaboration of relative abundance of each phenotype in the two groups, and the differences in absolute abundance need to be further confirmed.

There are several limitations in the study. First, considering the strong correlation between aging and OA, we selected healthy young people as the control group by 1:1 sex matching, for we could not exclude the possibility of asymptomatic elderly people suffering from early stage of OA. Meanwhile, the composition of GMB is also affected by aging (Paul and Ian, 2015). As a result, there were significant differences in age and BMI between the two groups. Although we corrected the two factors by MaAsLin, the results may also be affected by confounding factors. Therefore, studies with large samples and strictly matching are needed to verify the result. Second, the study only found the association between GMB and OA, and could not establish the causality. For this, longitudinal cohort studies are needed. Third, the study is only a comparison at the phenomenon level, so the underlying mechanism needs to be further investigated. Fourth, although 16S rRNA sequencing can recognize the classification and composition of GMB, it has limitations in identifying specific species and strains. Metagenomics is needed to explore the potential association between GMB and OA in the future.

In all, the study provides novel evidence that alterations of GMB in composition, function, and phenotype were observed in stool samples between OA patients and healthy people. The abundance of *Prevotella* significantly decreased in OA patients, indicating that it could be a potential diagnostic biomarker. In addition, the differences in the function and phenotype of GMB between the two populations may provide a novel perspective on further study in OA as well.

## Data availability statement

The data presented in the study are deposited in the SRA repository, accession number PRJNA853463.

## Ethics statement

The study was approved by the ethics committee of Peking University Shenzhen Hospital. The patients/participants provided their written informed consent to participate in this study.

## Author contributions

SL and GL designed and conducted the experiment. YZ and CX collected the stool samples. YZ, QY, and AX participated in the data analysis. JW and FY wrote the manuscript. HZ revised the manuscript. All authors contributed to the article and approved the submitted version.

## Funding

This research was continuously funded by National Natural Science Foundation of China (nos. 82172432, 82102568, 82001319, and 82102076), National and Local Joint Engineering Research Center of Orthopaedic Biomaterials (XMHT20190204007), Shenzhen High-level Hospital Construction Fund, Shenzhen Key Medical Discipline Construction Fund (no. SZXK023), Shenzhen “San-Ming” Project of Medicine (no. SZSM201612092), Research and Development Projects of Shenzhen (no. Z2021N054), Guangdong Basic and Applied Basic Research Foundation (nos. 2021A1515012586, 2019A1515011290, and 2019A1515110983), Bethune Charitable Foundation and CSPC Osteoporosis Research Foundation Project (no. G-X-2020-1107-21), and the Scientific Research Foundation of PEKING UNIVERSITY SHENZHEN HOSPITAL (no. KYQD2021099).

## Conflict of interest

The authors declare that the research was conducted in the absence of any commercial or financial relationships that could be construed as a potential conflict of interest.

## Publisher’s note

All claims expressed in this article are solely those of the authors and do not necessarily represent those of their affiliated organizations, or those of the publisher, the editors and the reviewers. Any product that may be evaluated in this article, or claim that may be made by its manufacturer, is not guaranteed or endorsed by the publisher.

## Footnotes

^1^ http://ccb.jhu.edu/software/FLASH/
