# Supplementary material for: Analysis of gut microbiome composition, function, and phenotype in patients with osteoarthritis
Source: Front Microbiol. 2022 Nov 25;13:980591. doi: 10.3389/fmicb.2022.980591 (PMC9732244; doi:10.3389/fmicb.2022.980591)
Supplement: Supplementary file 2 [file Data_Sheet_2.docx]

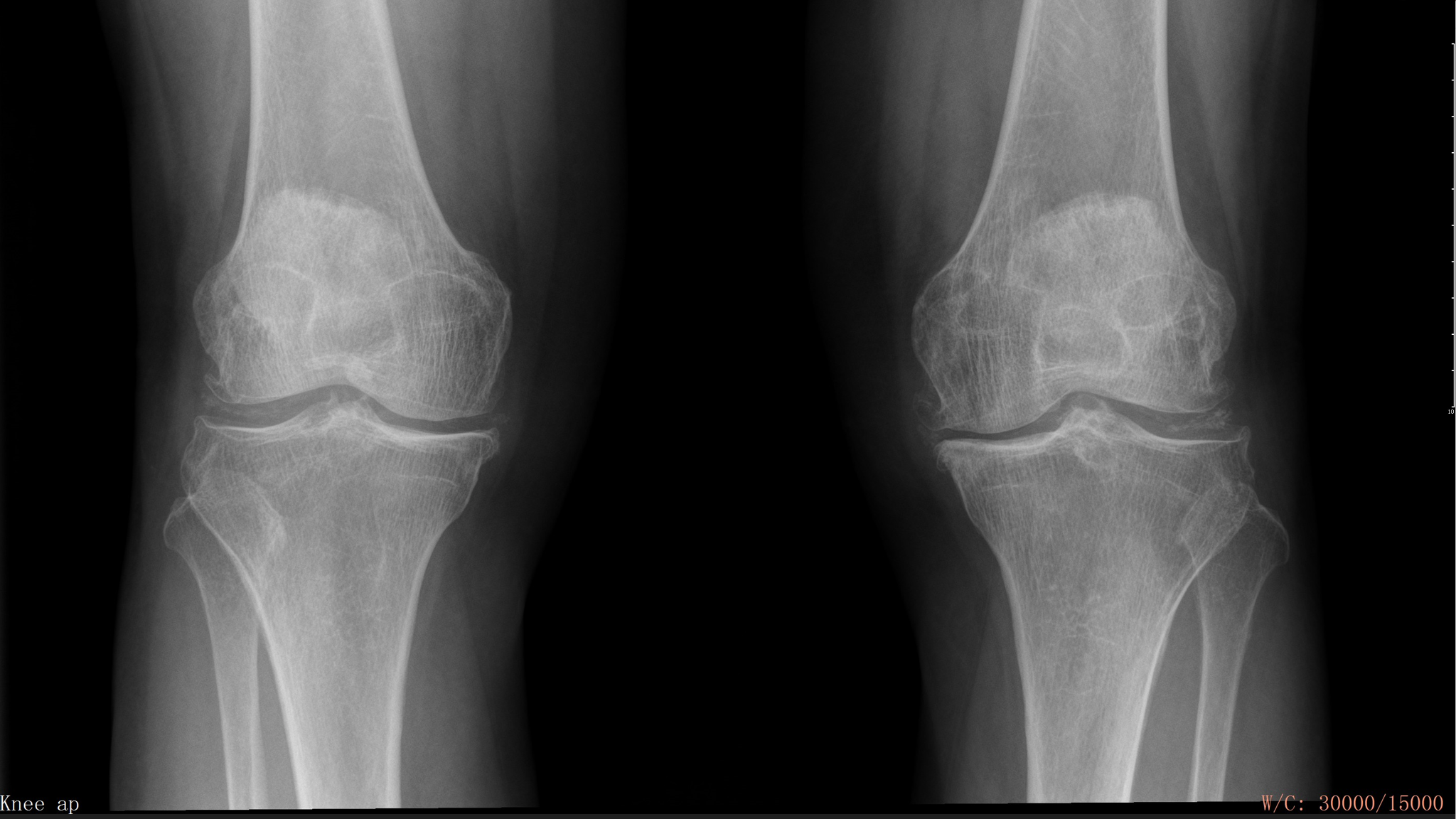


a


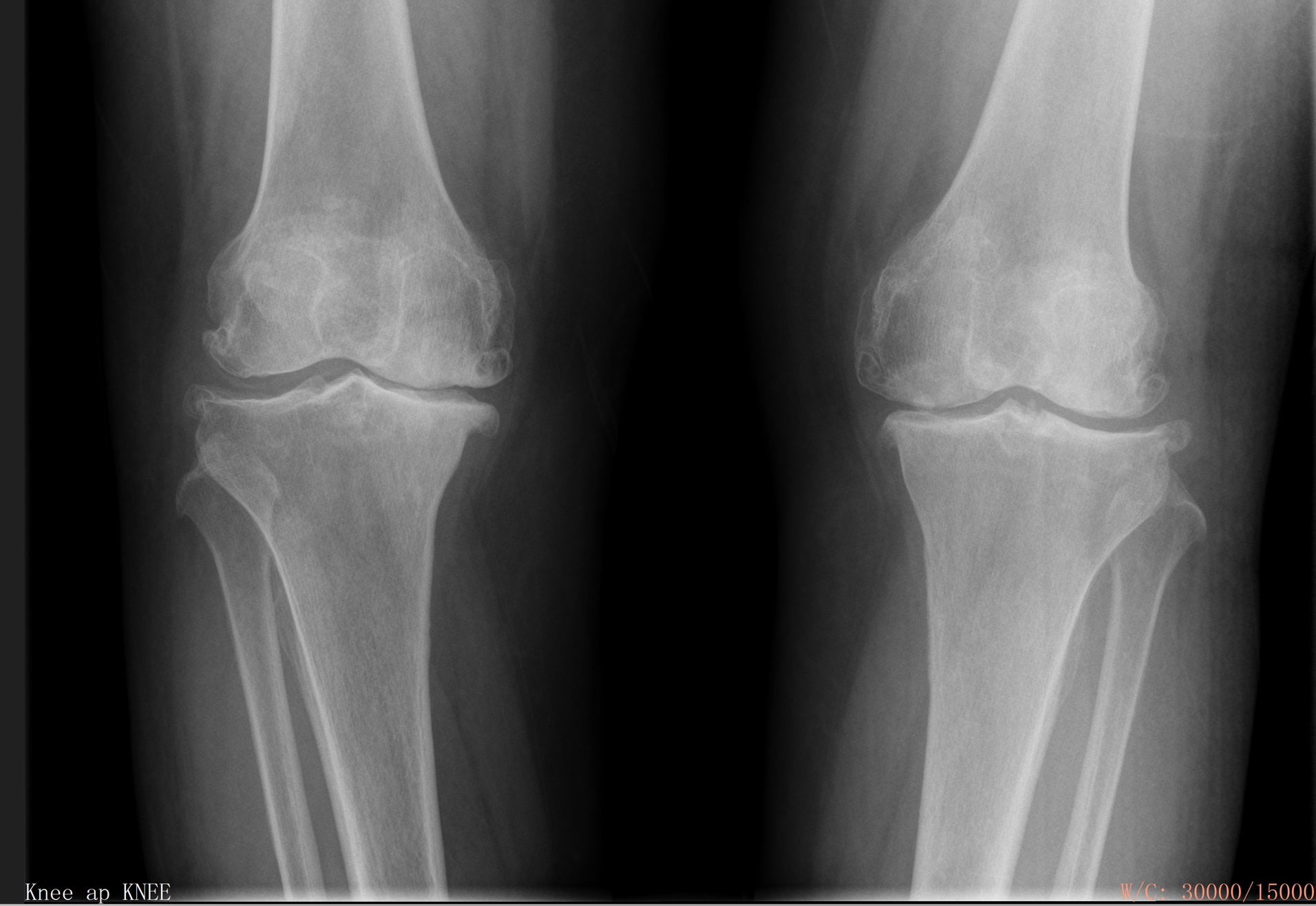


b

Figure S1. X-ray of knee in anterior-posterior position. a, Kellgren-Lawrence Ⅲ class: Moderate joint space narrowing. b, Kellgren-Lawrence Ⅳ class: Severe joint space narrowing, accompanied with subchondral sclerosis.


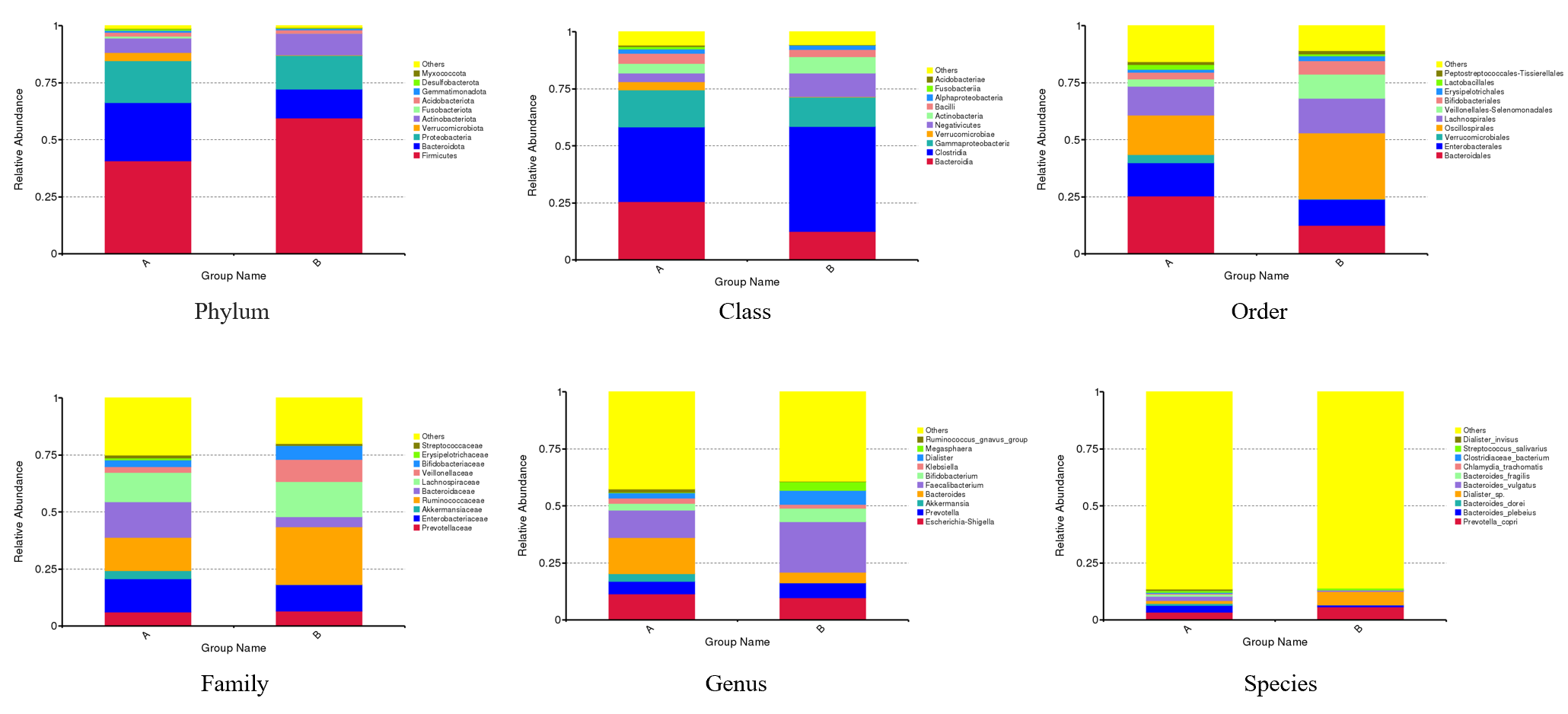


Figure S2. Histogram of relative abundance between two groups at six levels. The horizontal axis represents different groups and the vertical axis represents relative abundance. A color represents a species. Others represents all species other than the top 10. A, osteoarthritis group; B, healthy control group.


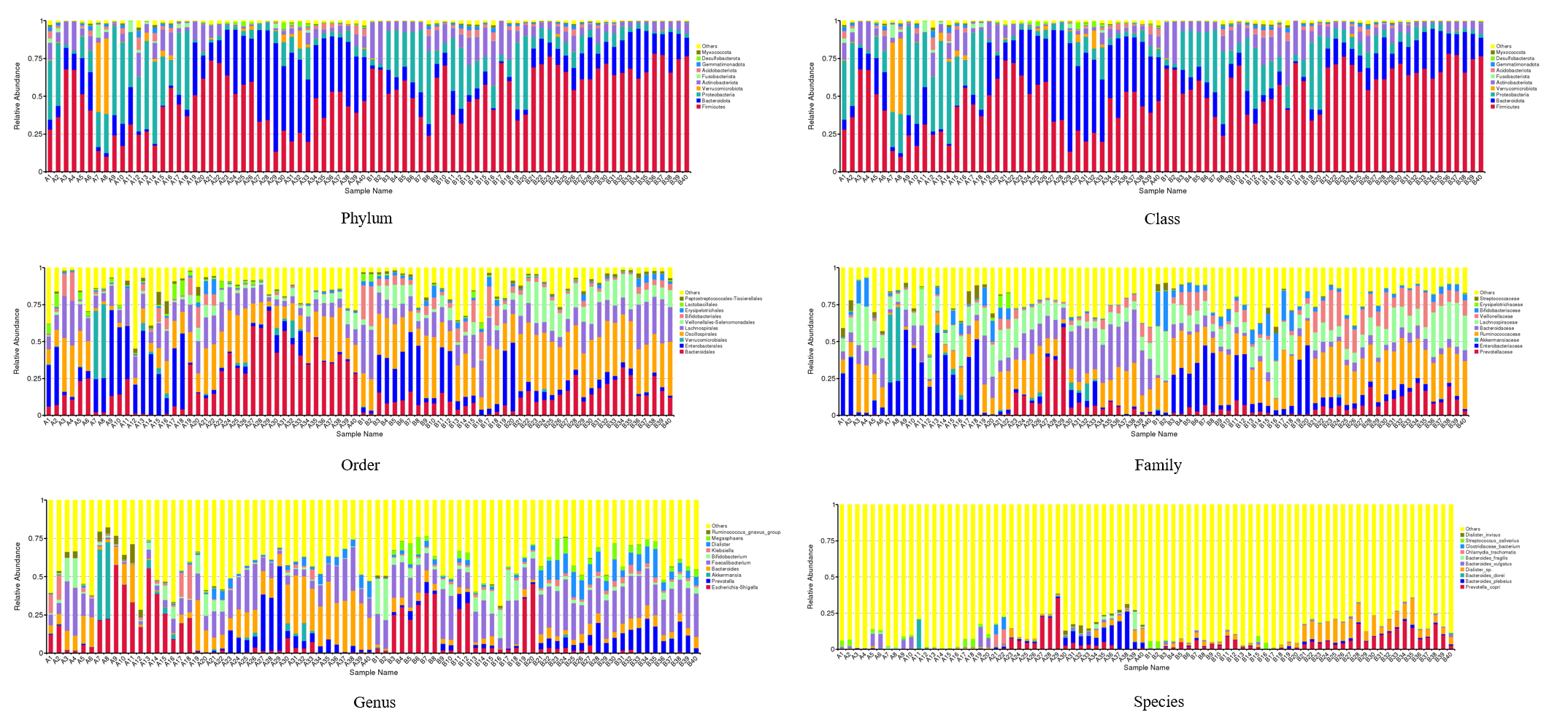


Figure S3. Histogram of relative abundance in all samples at six levels. The horizontal axis represents different groups and the vertical axis represents relative abundance. A color represents a species. Others represents all species other than the top 10. A, osteoarthritis group; B, healthy control group.


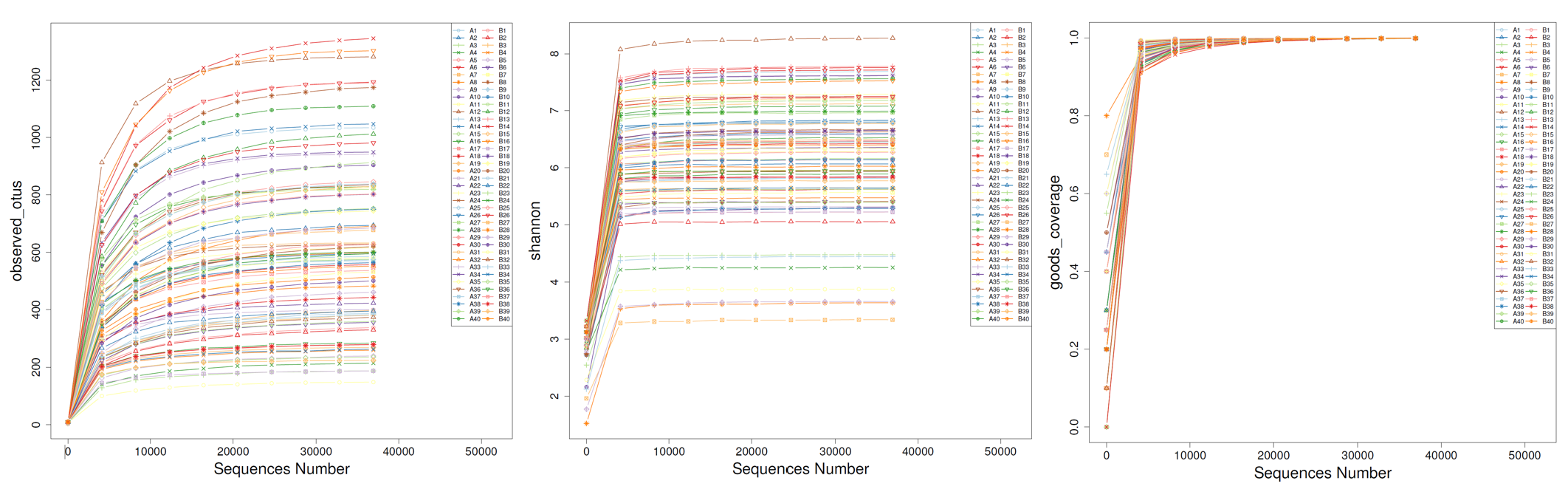


Figure S4. Rarefaction Curves. The horizontal axis represents the amount of sequencing data, and the vertical axis represents the corresponding alpha diversity index. When the curve tended to be flat, it indicated that the sequencing data amount was progressive and reasonable, and more data would not have a significant impact on alpha diversity index. Different colored curves represent different samples.


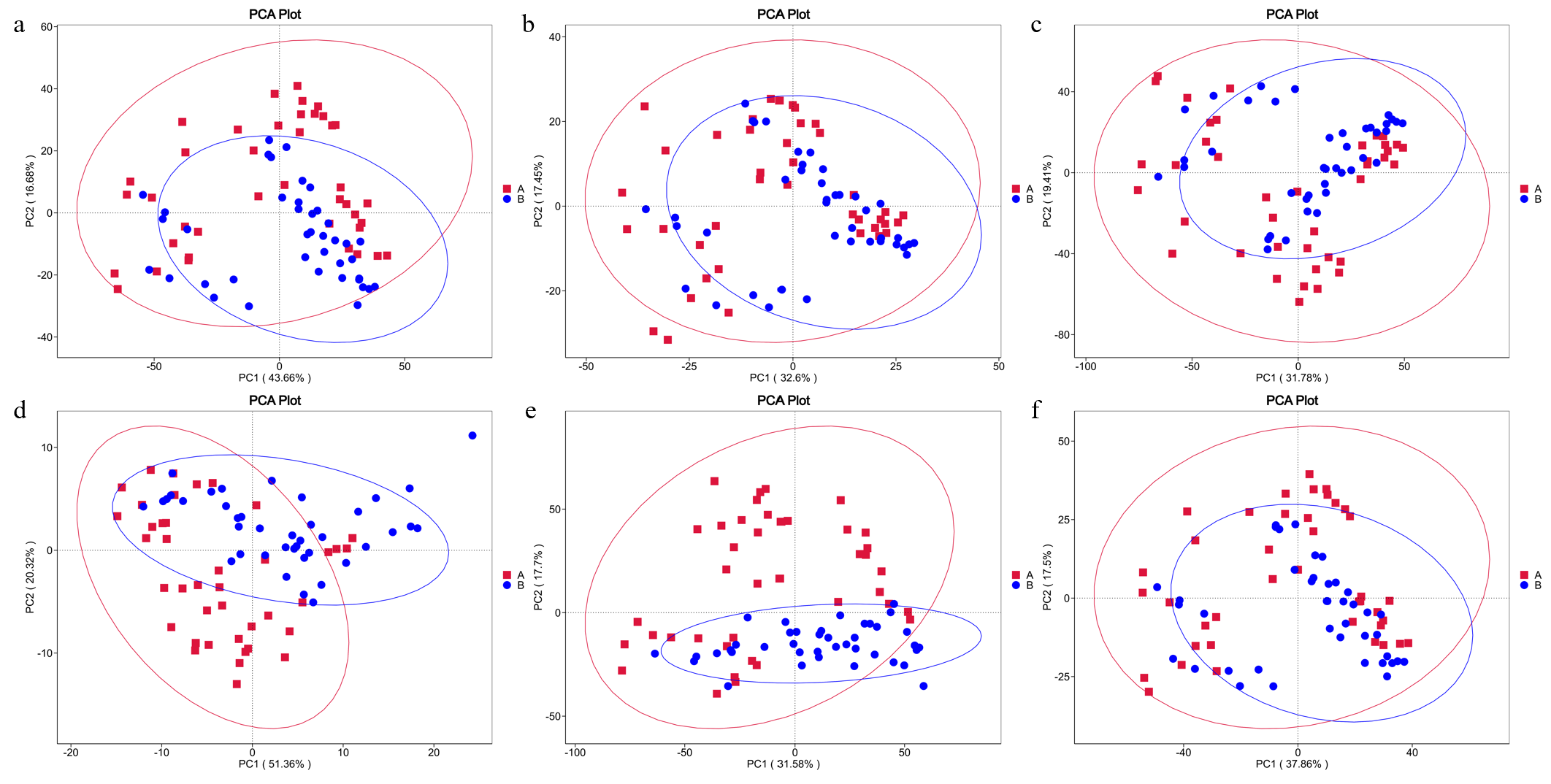


Figure S5. PCA Plots of predicted function. a, Clusters of Orthologous Genes (COGs); b, Enzyme Nomenclature; c, KEGG Orthology Functional Orthologues (KO); d, Kyoto Encyclopedia of Genes and Genomes (KEGG); e, Protein Families (PFAM); f, Protein Families Featuring Curated Multiple Sequence Alignments (TIGRFAM). The horizonal axis and vertical axis represent different principal components. Percentage represents the contribution of principal component to sample difference. Each dot represents a sample, and samples from the same group are shown in the same color. A, osteoarthritis group; B, healthy control group.


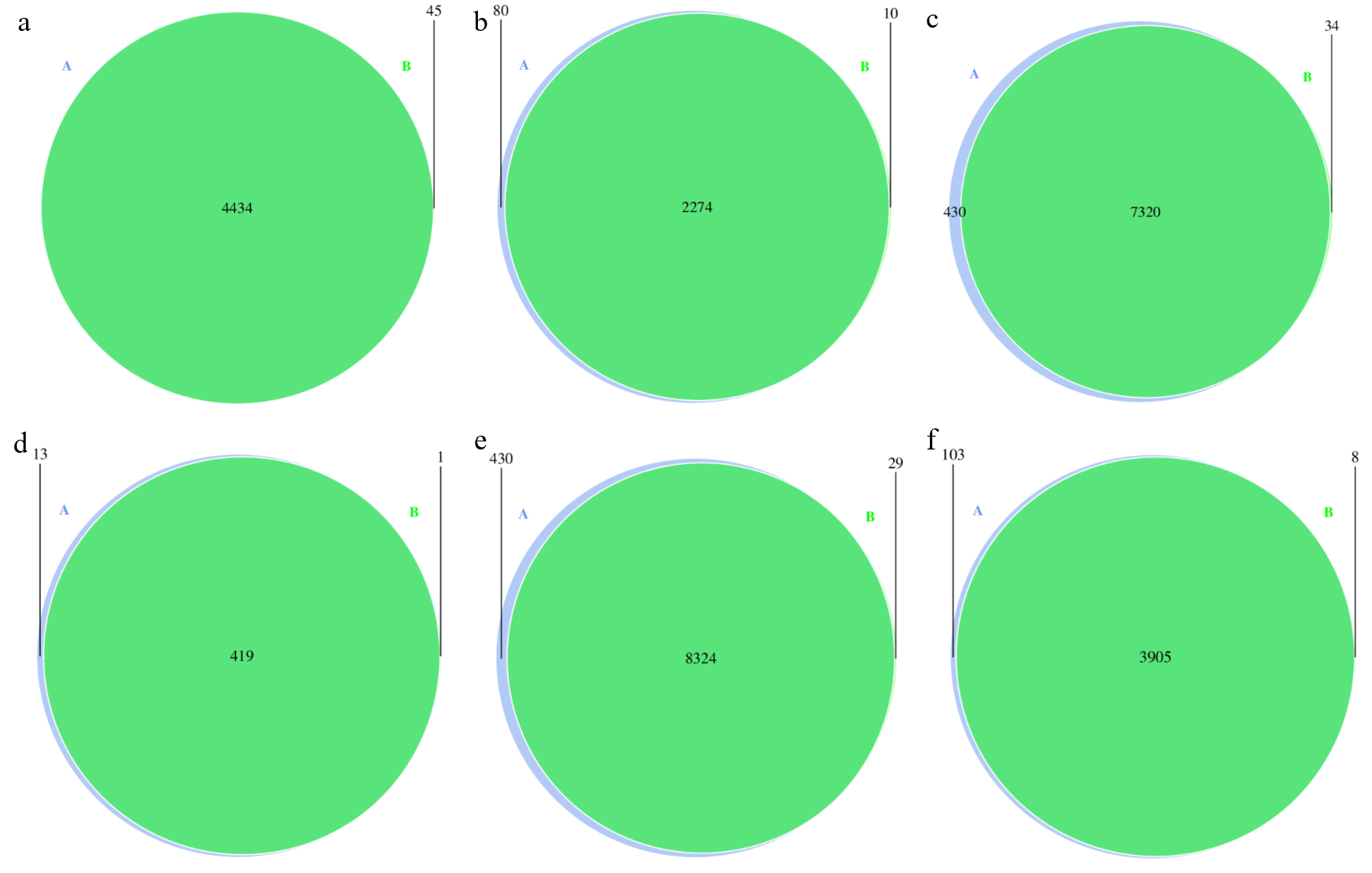


Figure S6. Venn diagram of predicted function. a, Clusters of Orthologous Genes (COGs); b, Enzyme Nomenclature; c, KEGG Orthology Functional Orthologues (KO); d, Kyoto Encyclopedia of Genes and Genomes (KEGG); e, Protein Families (PFAM); f, Protein Families Featuring Curated Multiple Sequence Alignments (TIGRFAM). Each circle in the diagram represents a group of samples. The overlapped circles represent the number of funtions shared by two groups, and the unoverlapped represents the number of functions unique to each group. A, osteoarthritis group; B, healthy control group.
