# Supplementary material for: Analysis of gut microbiome composition, function, and phenotype in patients with osteoarthritis
Source: Front Microbiol. 2022 Nov 25;13:980591. doi: 10.3389/fmicb.2022.980591 (PMC9732244; doi:10.3389/fmicb.2022.980591)
Supplement: Supplementary file 3 [file Data_Sheet_3.docx]

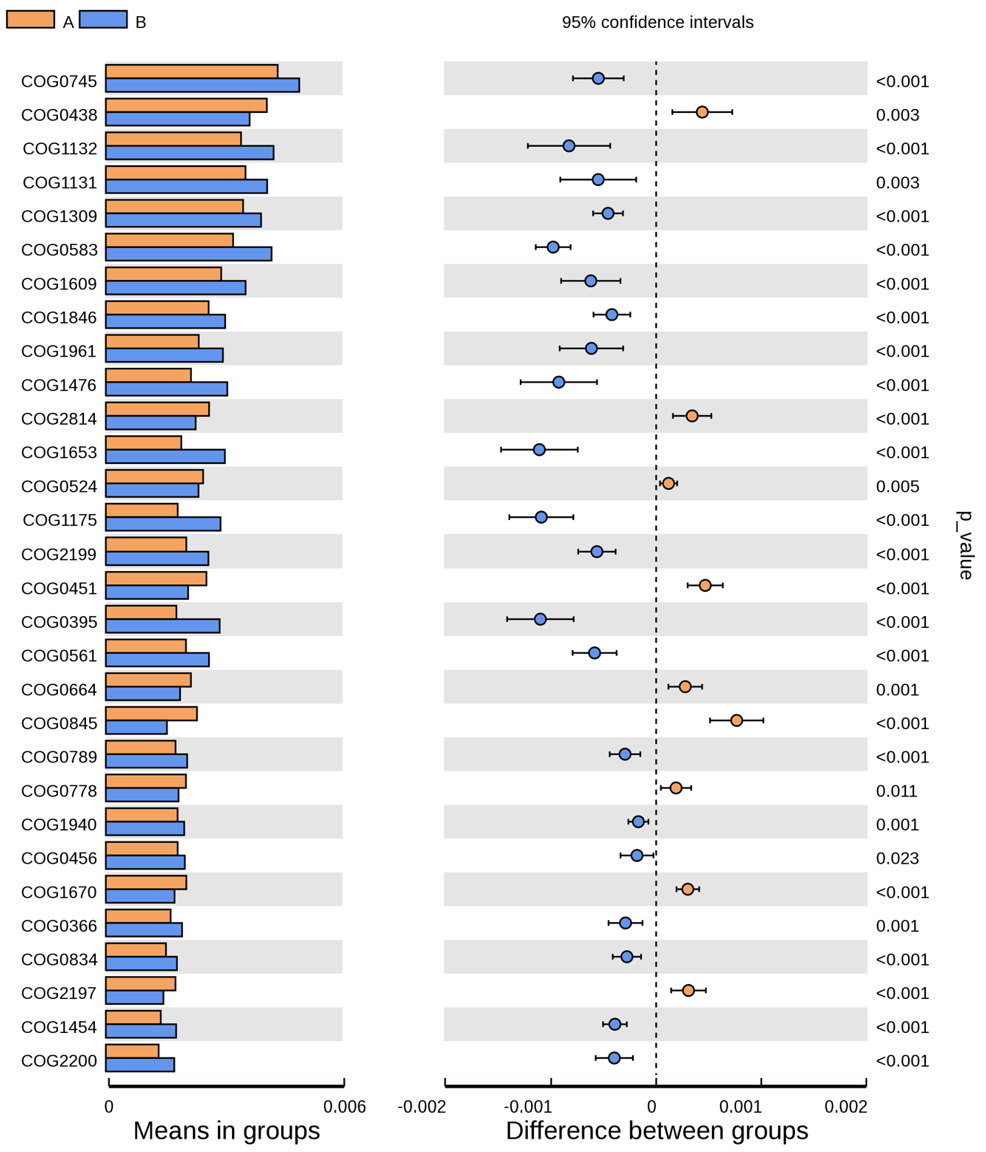


b

a

| Function | OA | Control | *P* | *Q* |
| --- | --- | --- | --- | --- |
| COG0745 | 201561^##^ | 221739^##^ | 0.002^*^ | **0.028**^&^ |
| COG0438 | 186265^##^ | 165148^##^ | 0.005^*^ | 0.277 |
| COG1132 | 154787±40404^#^ | 190840±34463^#^ | <0.001^**^ | 0.690 |
| COG1131 | 159881±38051^#^ | 183841±33266^#^ | 0.004^*^ | 0.525 |
| COG1309 | 159156±30575^#^ | 178693±22198^#^ | 0.002^*^ | **0.029**^&^ |
| COG0583 | 147960^##^ | 190206^##^ | <0.001^**^ | **0.038**^&^ |
| COG1609 | 133086±33651^#^ | 160458±29401^#^ | <0.001^**^ | 0.180 |
| COG1846 | 118901±25409^#^ | 136520±17378^#^ | 0.001^*^ | 0.076 |
| COG1961 | 106500±31076^#^ | 132721±23267^#^ | <0.001^**^ | 0.612 |
| COG1476 | 97004±34577^#^ | 137460±32785^#^ | <0.001^**^ | 0.750 |
| COG2814 | 120424±30219^#^ | 105038±30318^#^ | 0.026^*^ | **0.025**^&^ |
| COG1653 | 87216±37397^#^ | 135535±36057^#^ | <0.001^**^ | 0.999 |
| COG0524 | 112542±20410^#^ | 107054±16064^#^ | 0.185 | — |
| COG1175 | 83421±32506^#^ | 130897±30345^#^ | <0.001^**^ | 0.842 |
| COG2199 | 92445^##^ | 126548^##^ | <0.001^**^ | 0.059 |
| COG0451 | 113247^##^ | 92398^##^ | <0.001^**^ | **0.034**^&^ |
| COG0395 | 81730±32618^#^ | 129605±31234^#^ | <0.001^**^ | 0.991 |
| COG0561 | 92066±23704^#^ | 117731±18337^#^ | <0.001^**^ | 0.249 |
| COG0664 | 97256±20651^#^ | 85023±7352^#^ | 0.001^*^ | 0.279 |
| COG0845 | 104367±32165^#^ | 70610±21491^#^ | <0.001^**^ | 0.173 |
| COG0789 | 81095±21337^#^ | 93593±17057 | 0.005^*^ | 0.175 |
| COG0778 | 91052±15669^#^ | 82993±4871^#^ | 0.003^*^ | 0.452 |
| COG1940 | 82945±14889^#^ | 90506±15284^#^ | 0.028^*^ | 0.160 |
| COG0456 | 82939^##^ | 86930^##^ | 0.154 | — |
| COG1670 | 94183^##^ | 80466^##^ | <0.001^**^ | **0.006**^&^ |
| COG0366 | 74884±18393^#^ | 87639±18476^#^ | 0.003^*^ | 0.156 |
| COG0834 | 62511^##^ | 80843^##^ | 0.008^*^ | **0.046**^&^ |
| COG2197 | 82243^##^ | 59843^##^ | 0.018^*^ | **0.020**^&^ |
| COG1454 | 63428±13994^#^ | 80149±9192^#^ | <0.001^**^ | 0.306 |
| COG2200 | 53355^##^ | 79808^##^ | 0.001^*^ | 0.058 |

COG: Predicted function (Top 30) of gut microbiota based on COG analysis. a, the histogram of relative abundance; b, the table of absolute abundance. COG0745: DNA-binding response regulator, OmpR family, contains REC and winged-helix (wHTH) domain; COG0438: Glycosyltransferase involved in cell wall biosynthesis; COG1132: ABC-type multidrug transport system, ATPase and permease component; COG1131: ABC-type multidrug transport system, ATPase component; COG1309: DNA-binding transcriptional regulator, AcrR family; COG0583: DNA-binding transcriptional regulator, LysR family; COG1609: DNA-binding transcriptional regulator, LacI/PurR family; COG1846: DNA-binding transcriptional regulator, MarR family; COG1961: Site-specific DNA recombinase related to the DNA invertase Pin; COG1476: DNA-binding transcriptional regulator, XRE-family HTH domain; COG2814: Predicted arabinose efflux permease, MFS family; COG1653: ABC-type glycerol-3-phosphate transport system, periplasmic component; COG0524: Sugar or nucleoside kinase, ribokinase family; COG1175: ABC-type sugar transport system, permease component; COG2199: GGDEF domain, diguanylate cyclase (c-di-GMP synthetase) or its enzymatically inactive variants; COG0451: Nucleoside-diphosphate-sugar epimerase; COG0395: ABC-type glycerol-3-phosphate transport system, permease component; COG0561: Hydroxymethylpyrimidine pyrophosphatase and other HAD family phosphatases; COG0664: cAMP-binding domain of CRP or a regulatory subunit of cAMP-dependent protein kinases; COG0845: Multidrug efflux pump subunit AcrA (membrane-fusion protein); COG0789: DNA-binding transcriptional regulator, MerR family; COG0778: Nitroreductase; COG1940: Sugar kinase of the NBD/HSP70 family, may contain an N-terminal HTH domain; COG0456: Ribosomal protein S18 acetylase RimI and related acetyltransferases; COG1670: Protein N-acetyltransferase, RimJ/RimL family; COG0366: Glycosidase; COG0834: ABC-type amino acid transport/signal transduction system, periplasmic component/domain; COG2197: DNA-binding response regulator, NarL/FixJ family, contains REC and HTH domains; COG1454: Alcohol dehydrogenase, class IV; COG2200: EAL domain, c-di-GMP-specific phosphodiesterase class I (or its enzymatically inactive variant). A, osteoarthritis group; B, healthy control group; OA, osteoarthritis; *Q*, *P* value after adjusting for gender, age, and body mass index (BMI). #, mean ± standard deviation; ##, median. *, *P* < 0.05, statistical difference; **, *P* < 0.001, dramatically statistical difference; &, Q < 0.05, statistical difference.


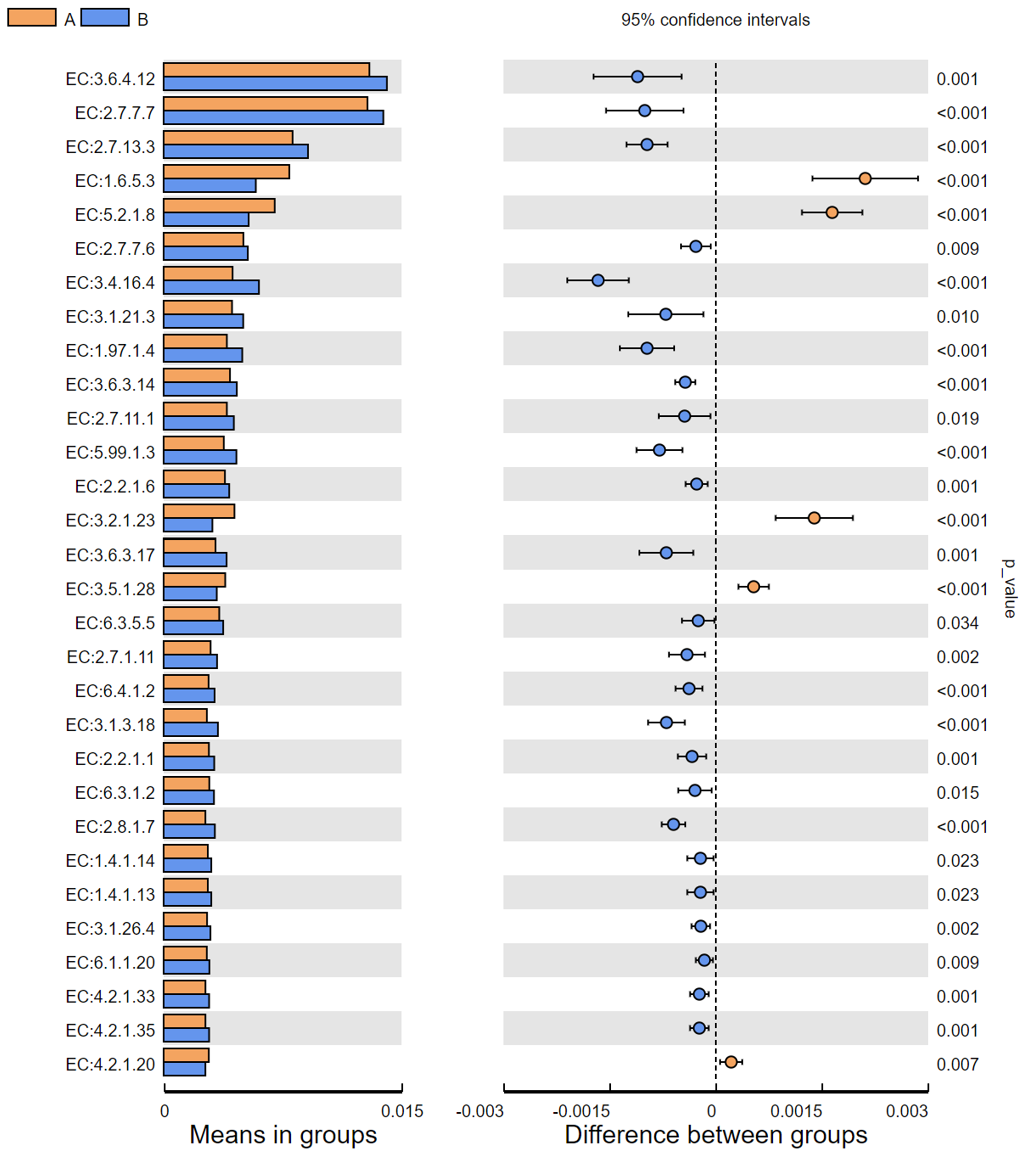


b

a

| Function | OA | Control | *P* | *Q* |
| --- | --- | --- | --- | --- |
| EC:3.6.4.12 | 246601±31374^#^ | 262662±19157^#^ | 0.011^*^ | 0.233 |
| EC:2.7.7.7 | 244504^##^ | 254406^##^ | 0.044^*^ | 0.140 |
| EC:2.7.13.3 | 156797±30898^#^ | 170494±20685^#^ | 0.022^*^ | **0.004**^&^ |
| EC:1.6.5.3 | 151585±41821^#^ | 109838±33725^#^ | <0.001^**^ | 0.167 |
| EC:5.2.1.8 | 133065±23221^#^ | 100596±17570^#^ | <0.001^**^ | 0.119 |
| EC:2.7.7.6 | 95721±12583^#^ | 98851±7996^#^ | 0.188 | — |
| EC:3.4.16.4 | 83141±20788^#^ | 111338±14696^#^ | <0.001^**^ | 0.633 |
| EC:3.1.21.3 | 79431^##^ | 96017^##^ | 0.046^*^ | 0.610 |
| EC:1.97.1.4 | 75890±18278^#^ | 91833±12266 | <0.001^**^ | 0.655 |
| EC:3.6.3.14 | 77826^##^ | 87594^##^ | 0.032^*^ | **0.017**^&^ |
| EC:2.7.11.1 | 776947±21794^#^ | 82577±15758^#^ | 0.189 | — |
| EC:5.99.1.3 | 72279±15506^#^ | 85143±11608^#^ | <0.001^**^ | 0.667 |
| EC:2.2.1.6 | 74356±14255^#^ | 77558±10744^#^ | 0.260 | — |
| EC:3.2.1.23 | 84287±29910^#^ | 56854±12441^#^ | <0.001^**^ | 0.593 |
| EC:3.6.3.17 | 54789^##^ | 77270^##^ | 0.021^*^ | **0.034**^&^ |
| EC:3.5.1.28 | 74091±12058^#^ | 62421±8864^#^ | <0.001^**^ | **0.002**^&^ |
| EC:6.3.5.5 | 68586^##^ | 69100^##^ | 0.317 | — |
| EC:2.7.1.11 | 56402±11509^#^ | 62310±8589^#^ | 0.011^*^ | 0.334 |
| EC:6.4.1.2 | 54984±14461^#^ | 60258±9867^#^ | 0.060 | — |
| EC:3.1.3.18 | 51448±9319^#^ | 63216±8769^#^ | <0.001^**^ | 0.452 |
| EC:2.2.1.1 | 54671±11447^#^ | 59216±6464^#^ | 0.032^*^ | **0.043**^&^ |
| EC:6.3.1.2 | 55009±12247^#^ | 58872±7593^#^ | 0.090 | — |
| EC:2.8.1.7 | 49425^##^ | 57983^##^ | <0.001^**^ | 0.241 |
| EC:1.4.1.14 | 54419^##^ | 53563^##^ | 0.408 | — |
| EC:1.4.1.13 | 54419^##^ | 53563^##^ | 0.408 | — |
| EC:3.1.26.4 | 52935^##^ | 54494^##^ | 0.128 | — |
| EC:6.1.1.20 | 51801±6752^#^ | 53656±4151^#^ | 0.143 | — |
| EC:4.2.1.33 | 50415±8702^#^ | 53461±5636^#^ | 0.067 | — |
| EC:4.2.1.35 | 50384±8693^#^ | 53439±5629^#^ | 0.066 | — |
| EC:4.2.1.20 | 54176±8711^#^ | 49009±4991^#^ | 0.002^*^ | 0.054 |

EC: Predicted function (Top 30) of gut microbiota based on EC analysis. a, the histogram of relative abundance; b, the table of absolute abundance. EC:3.6.4.12: DNA helicase; EC:2.7.7.7: DNA-directed DNA polymerase; EC:2.7.13.3: Histidine kinase; EC:1.6.5.3: NADH:ubiquinone reductase (H(+)-translocating); EC:5.2.1.8: Peptidylprolyl isomerase; EC:2.7.7.6: DNA-directed RNA polymerase; EC:3.4.16.4: Serine-type D-Ala-D-Ala carboxypeptidase; EC:3.1.21.3: Type I site-specific deoxyribonuclease; EC:1.97.1.4: [Formate-C-acetyltransferase]-activating enzyme; EC:3.6.3.14: H(+)-transporting two-sector ATPase; EC:2.7.11.1: Non-specific serine/threonine protein kinase; EC:5.99.1.3: DNA topoisomerase (ATP-hydrolyzing); EC:2.2.1.6: Acetolactate synthase; EC:3.2.1.23: Beta-galactosidase; EC:3.6.3.17: Monosaccharide-transporting ATPase; EC:3.5.1.28: N-acetylmuramoyl-L-alanine amidase; EC:6.3.5.5: Carbamoyl-phosphate synthase (glutamine-hydrolyzing); EC:2.7.1.11: 6-phosphofructokinase; EC:6.4.1.2: Acetyl-CoA carboxylase; EC:3.1.3.18: Phosphoglycolate phosphatase; EC:2.2.1.1: Transketolase; EC:6.3.1.2: Glutamate--ammonia ligase; EC:2.8.1.7: Cysteine desulfurase; EC:1.4.1.14: Glutamate synthase (NADH); EC:1.4.1.13: Glutamate synthase (NADPH); EC:3.1.26.4: Ribonuclease H; EC:6.1.1.20: Phenylalanine--tRNA ligase; EC:4.2.1.33: 3-isopropylmalate dehydratase; EC:4.2.1.35: (R)-2-methylmalate dehydratase; EC:4.2.1.20: Tryptophan synthase. A, osteoarthritis group; B, healthy control group; OA, osteoarthritis; *Q*, *P* value after adjusting for gender, age, and body mass index (BMI). #, mean ± standard deviation; ##, median. *, *P* < 0.05, statistical difference; **, *P* < 0.001, dramatically statistical difference; &, Q < 0.05, statistical difference.


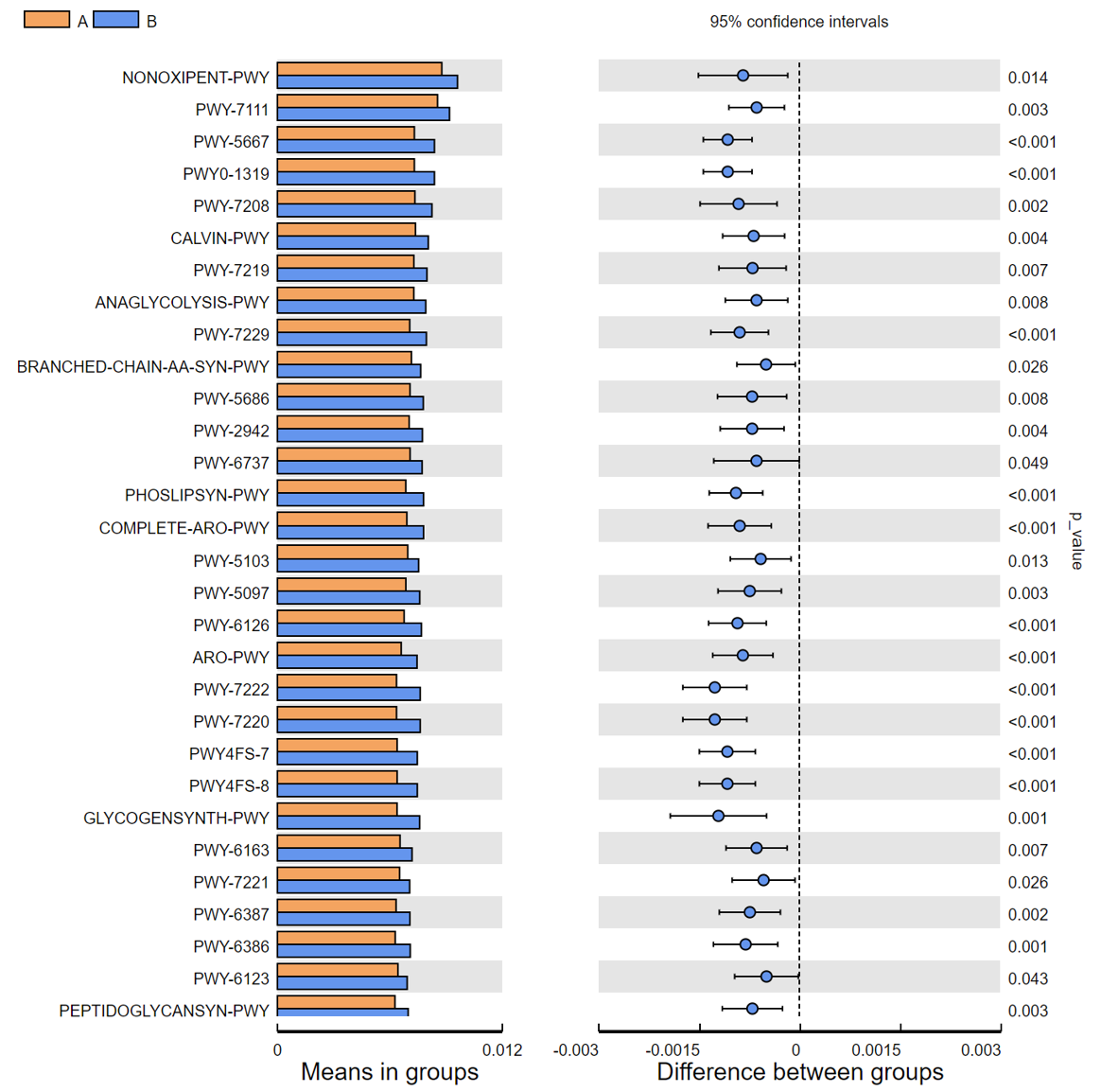


b

a

| Function | OA | Control | *P* | *Q* |
| --- | --- | --- | --- | --- |
| NONOXIPENT-PWY | 157807±41930^#^ | 185207±34796^#^ | 0.002^*^ | 0.579 |
| PWY-7111 | 148170±48456^#^ | 182768±41750^#^ | 0.001^*^ | 0.889 |
| PWY-5667 | 30834^##^ | 35316^##^ | 0.028^*^ | 0.396 |
| PWY0-1319 | 133363±41731^#^ | 157047±35956^#^ | 0.008^*^ | 0.814 |
| PWY-7208 | 31676^##^ | 33185^##^ | 0.046^*^ | 0.389 |
| CALVIN-PWY | 86162±23276^#^ | 108681±19092^#^ | <0.001^**^ | 0.433 |
| PWY-7219 | 59599±12948^#^ | 77211±11038^#^ | <0.001^**^ | 0.730 |
| ANAGLYCOLYSIS-PWY | 63074±12795^#^ | 72441±8636^#^ | <0.001^**^ | 0.910 |
| PWY-7229 | 48310±20456^#^ | 71161±18641^#^ | <0.001^**^ | 0.605 |
| BRANCHED-CHAIN-AA-SYN-PWY | 48203±14542^#^ | 70629±9332^#^ | <0.001^**^ | 0.682 |
| PWY-5686 | 51447±9320^#^ | 63216±8769^#^ | <0.001^**^ | 0.452 |
| PWY-2942 | 54670±11447^#^ | 59216±6464^#^ | 0.032^*^ | **0.043**^&^ |
| PWY-6737 | 48490±17400^#^ | 64841±12880^#^ | <0.001^**^ | 0.866 |
| PHOSLIPSYN-PWY | 53285±13071^#^ | 59387±10186^#^ | 0.022^*^ | 0.692 |
| COMPLETE-ARO-PWY | 68151±28639^#^ | 41173±9738^#^ | <0.001^**^ | 0.566 |
| PWY-5103 | 30745^##^ | 30916^##^ | 0.027^*^ | **0.010**^&^ |
| PWY-5097 | 42832±17046^#^ | 64387±15665^#^ | <0.001^**^ | 0.907 |
| PWY-6126 | 41714±16095^#^ | 62748±14871^#^ | <0.001^**^ | 0.941 |
| ARO-PWY | 48309±17520^#^ | 55521±9329^#^ | 0.024^*^ | 0.426 |
| PWY-7222 | 26782^##^ | 30997^##^ | 0.058 | — |
| PWY-7220 | 53281±9737^#^ | 46816±6746^#^ | 0.001^*^ | **0.002**^&^ |
| PWY4FS-7 | 26652^##^ | 30609^##^ | <0.001^**^ | **0.019**^&^ |
| PWY4FS-8 | 26652^##^ | 30609^##^ | <0.001^**^ | 0.098 |
| GLYCOGENSYNTH-PWY | 42642±10527^#^ | 53047±9258^#^ | <0.001^**^ | 0.866 |
| PWY-6163 | 27951^##^ | 28932^##^ | <0.001^**^ | 0.916 |
| PWY-7221 | 44216±9460^#^ | 49805±7074^#^ | 0.004^*^ | 0.779 |
| PWY-6387 | 42475±8139^#^ | 50920±6736^#^ | <0.001^**^ | 0.647 |
| PWY-6386 | 50507±16928^#^ | 41701±6868^#^ | 0.003^*^ | 0.799 |
| PWY-6123 | 28144^##^ | 27961^##^ | 0.009^*^ | 0.177 |
| PEPTIDOGLYCANSYN-PWY | 59974±23287^#^ | 31546±12014^#^ | <0.001^**^ | 0.524 |

KEGG: Predicted function (Top 30) of gut microbiota based on KEGG analysis. a, the histogram of relative abundance; b, the table of absolute abundance. NONOXIPENT-PWY: pentose phosphate pathway (non-oxidative branch); PWY-7111: pyruvate fermentation to isobutanol (engineered); PWY-5667: CDP-diacylglycerol biosynthesis I; PWY0-1319: CDP-diacylglycerol biosynthesis II; PWY-7208: superpathway of pyrimidine nucleobases salvage; CALVIN-PWY: Calvin-Benson-Bassham cycle; PWY-7219: adenosine ribonucleotides de novo biosynthesis; ANAGLYCOLYSIS-PWY: glycolysis III (from glucose); PWY-7229: superpathway of adenosine nucleotides de novo biosynthesis I; BRANCHED-CHAIN-AA-SYN-PWY: superpathway of branched amino acid biosynthesis; PWY-5686: UMP biosynthesis; PWY-2942: L-lysine biosynthesis III; PWY-6737: starch degradation V; PHOSLIPSYN-PWY: superpathway of phospholipid biosynthesis I (bacteria); PWY-5103: L-isoleucine biosynthesis III; PWY-5097: L-lysine biosynthesis VI; PWY-6126: superpathway of adenosine nucleotides de novo biosynthesis II; ARO-PWY: chorismate biosynthesis I; PWY-7222: guanosine deoxyribonucleotides de novo biosynthesis II; PWY-7220: adenosine deoxyribonucleotides de novo biosynthesis II; PWY4FS-7: phosphatidylglycerol biosynthesis I (plastidic); PWY4FS-8: phosphatidylglycerol biosynthesis II (non-plastidic); GLYCOGENSYNTH-PWY: glycogen biosynthesis I (from ADP-D-Glucose); PWY-6163: chorismate biosynthesis from 3-dehydroquinate; PWY-7221: guanosine ribonucleotides de novo biosynthesis; PWY-6387: UDP-N-acetylmuramoyl-pentapeptide biosynthesis I (meso-diaminopimelate containing); PWY-6386: UDP-N-acetylmuramoyl-pentapeptide biosynthesis II (lysine-containing); PWY-6123: inosine-5'-phosphate biosynthesis I; PEPTIDOGLYCANSYN-PWY: peptidoglycan biosynthesis I (meso-diaminopimelate containing). A, osteoarthritis group; B, healthy control group; OA, osteoarthritis; *Q*, *P* value after adjusting for gender, age, and body mass index (BMI). #, mean ± standard deviation; ##, median. *, *P* < 0.05, statistical difference; **, *P* < 0.001, dramatically statistical difference; &, Q < 0.05, statistical difference.


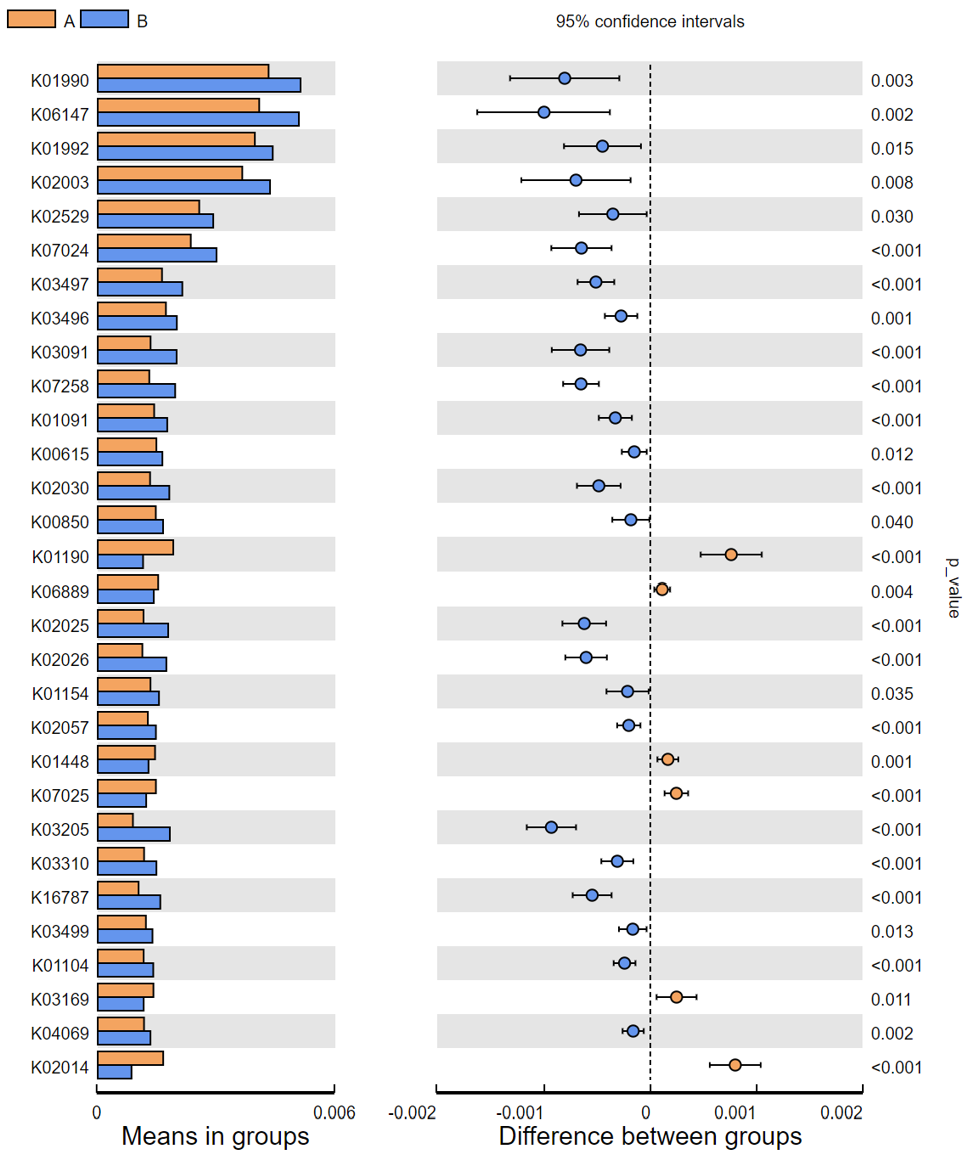


b

a

| Function | OA | Control | *P* | *Q* |
| --- | --- | --- | --- | --- |
| K01990 | 157807±41930^#^ | 185207±34796^#^ | 0.002^*^ | 0.579 |
| K06147 | 148170±48456^#^ | 182768±41750^#^ | 0.001^*^ | 0.889 |
| K01992 | 152330^##^ | 155572^##^ | 0.028^*^ | 0.396 |
| K02003 | 133363±41731^#^ | 157047±35956^#^ | 0.008^*^ | 0.814 |
| K02529 | 91152^##^ | 100145^##^ | 0.046^*^ | 0.389 |
| K07024 | 86162±23276^#^ | 108682±19092^#^ | <0.001^**^ | 0.433 |
| K03497 | 59599±12948^#^ | 77211±11038^#^ | <0.001^**^ | 0.730 |
| K03496 | 63074±12795^#^ | 72441±8636^#^ | <0.001^**^ | 0.910 |
| K03091 | 48310±20456^#^ | 71161±18641^#^ | <0.001^**^ | 0.605 |
| K07258 | 48203±14542^#^ | 70629±9332^#^ | <0.001^**^ | 0.682 |
| K01091 | 51447±9320^#^ | 63216±8769^#^ | <0.001^**^ | 0.452 |
| K00615 | 54670±11447^#^ | 59216±6464^#^ | 0.032^*^ | **0.043**^&^ |
| K02030 | 48490±17400^#^ | 64841±12880^#^ | <0.001^**^ | 0.866 |
| K00850 | 53285±13071^#^ | 59387±10186^#^ | 0.022^*^ | 0.692 |
| K01190 | 68151±28639^#^ | 41173±9738^#^ | <0.001^**^ | 0.566 |
| K06889 | 57086^##^ | 49012^##^ | 0.027^*^ | **0.010**^&^ |
| K02025 | 42832±17046^#^ | 64388±15665^#^ | <0.001^**^ | 0.907 |
| K02026 | 41714±16095^#^ | 62748±14871^#^ | <0.001^**^ | 0.941 |
| K01154 | 483009±17520^#^ | 55521±9329^#^ | 0.024^*^ | 0.426 |
| K02057 | 44549^##^ | 58618^##^ | 0.058 | — |
| K01448 | 53281±9737^#^ | 46816±6746^#^ | 0.001^*^ | **0.002**^&^ |
| K07025 | 53809^##^ | 46720^##^ | <0.001^**^ | **0.019**^&^ |
| K03205 | 29457^##^ | 69771^##^ | <0.001^**^ | 0.098 |
| K03310 | 42642±10527^#^ | 53047±9258^#^ | <0.001^**^ | 0.866 |
| K16787 | 36131^##^ | 56341^##^ | <0.001^**^ | 0.916 |
| K03499 | 44216±9460^#^ | 49805±7074^#^ | 0.004^*^ | 0.779 |
| K01104 | 42475±8139^#^ | 50920±6736^#^ | <0.001^**^ | 0.647 |
| K03169 | 50507±16928^#^ | 41701±6868^#^ | 0.003^*^ | 0.799 |
| K04069 | 41073^##^ | 47336^##^ | 0.009^*^ | 0.177 |
| K02014 | 59974±23287^#^ | 31546±12014^#^ | <0.001^**^ | 0.524 |

KO: Predicted function (Top 30) of gut microbiota based on KO analysis. a, the histogram of relative abundance; b, the table of absolute abundance. K01990: ABC-2 type transport system ATP-binding protein; K06147: ATP-binding cassette, subfamily B, bacterial; K01992: ABC-2 type transport system permease protein; K02003: putative ABC transport system ATP-binding protein; K02529: LacI family transcriptional regulator; K07024: sucrose-6-phosphatase; K03497: chromosome partitioning protein, ParB family; K03496: chromosome partitioning protein; K03091: RNA polymerase sporulation-specific sigma factor; K07258: serine-type D-Ala-D-Ala carboxypeptidase (penicillin-binding protein 5/6); K01091: phosphoglycolate phosphatase; K00615: transketolase; K02030: polar amino acid transport system substrate-binding protein; K00850: 6-phosphofructokinase 1; K01190: beta-galactosidase; K06889: uncharacterized protein; K02025: ABC.MS.P, multiple sugar transport system permease protein; K02026: ABC.MS.P1, multiple sugar transport system permease protein; K01154: type I restriction enzyme, S subunit; K02057: simple sugar transport system permease protein; K01448: N-acetylmuramoyl-L-alanine amidase; K07025: putative hydrolase of the HAD superfamily; K03205: type IV secretion system protein VirD4; K03310: alanine or glycine:cation symporter, AGCS family; K16787: energy-coupling factor transport system ATP-binding protein; K03499: trk system potassium uptake protein; K01104: protein-tyrosine phosphatase; K03169: DNA topoisomerase III; K04069: pyruvate formate lyase activating enzyme; K02014: iron complex outermembrane recepter protein. A, osteoarthritis group; B, healthy control group; OA, osteoarthritis; *Q*, *P* value after adjusting for gender, age, and body mass index (BMI). #, mean ± standard deviation; ##, median. *, *P* < 0.05, statistical difference; **, *P* < 0.001, dramatically statistical difference; &, Q < 0.05, statistical difference.


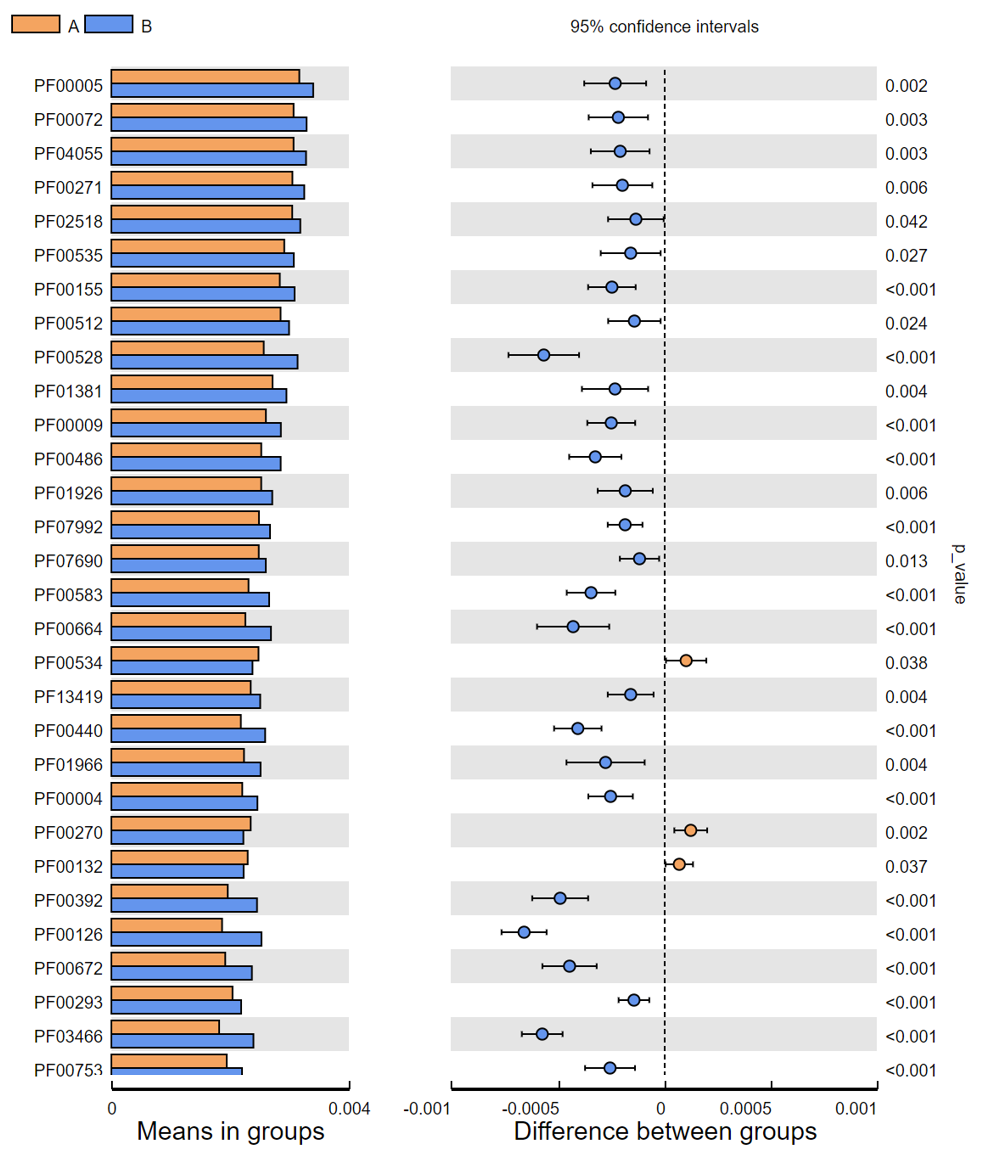


b

a

| Function | OA | Control | *P* | *Q* |
| --- | --- | --- | --- | --- |
| PF00005 | 256349±32984^#^ | 267698±20669^#^ | 0.069 | — |
| PF00072 | 253122^##^ | 256966^##^ | 0.436 | — |
| PF04055 | 256846^##^ | 256466^##^ | 0.381 | — |
| PF00271 | 253822^##^ | 252326^##^ | 0.317 | — |
| PF02518 | 252614^##^ | 249755^##^ | 0.977 | — |
| PF00535 | 235993±31868^#^ | 242287±25457^#^ | 0.332 | — |
| PF00155 | 237597^##^ | 241521^##^ | 0.072 | — |
| PF00512 | 241305^##^ | 234000^##^ | 0.878 | — |
| PF00528 | 209249±43413^#^ | 247497±26496^#^ | <0.001^**^ | 0.073 |
| PF01381 | 226725^##^ | 231921^##^ | 0.128 | — |
| PF00009 | 214123^##^ | 223004^##^ | 0.020^*^ | 0.120 |
| PF00486 | 206948^##^ | 224016^##^ | 0.020^*^ | **0.015**^&^ |
| PF01926 | 204348±27399^#^ | 213118±16181^#^ | 0.085 | — |
| PF07992 | 203000±31881^#^ | 211665±24883^#^ | 0.179 | — |
| PF07690 | 201509±27189^#^ | 205691±20852^#^ | 0.442 | — |
| PF00583 | 188191±31707^#^ | 210103±24684^#^ | 0.001^*^ | 0.064 |
| PF00664 | 183762±37393^#^ | 211720±28852^#^ | <0.001^**^ | 0.168 |
| PF00534 | 201035±27455^#^ | 188176±21392^#^ | 0.022^*^ | **0.046**^&^ |
| PF13419 | 191666^##^ | 199987^##^ | 0.326 | — |
| PF00440 | 178218±32430^#^ | 205065±26636^#^ | <0.001^**^ | 0.076 |
| PF01966 | 188891^##^ | 194397^##^ | 0.070 | — |
| PF00004 | 179283±27841^#^ | 193840±16727^#^ | 0.006^*^ | 0.160 |
| PF00270 | 190089±22458^#^ | 175832±15530^#^ | 0.001^*^ | 0.066 |
| PF00132 | 189164^##^ | 181314^##^ | 0.007^*^ | **0.003**^&^ |
| PF00392 | 160089±34380^#^ | 193868±2330^#^ | <0.001^**^ | 0.078 |
| PF00126 | 152705±31617^#^ | 199362±17108^#^ | <0.001^**^ | 0.060 |
| PF00672 | 157948±20604^#^ | 187811±26553^#^ | <0.001^**^ | 0.070 |
| PF00293 | 166708^##^ | 179595^##^ | 0.308 | — |
| PF03466 | 151293^##^ | 191960^##^ | <0.001^**^ | **0.034**^&^ |
| PF00753 | 158340±30360^#^ | 173398±19690^#^ | 0.010^*^ | 0.080 |

PFAM: Predicted function (Top 30) of gut microbiota based on PFAM analysis. a, the histogram of relative abundance; b, the table of absolute abundance. PF00005: ABC transporter; PF00072: Response regulator receiver domain; PF04055: Radical SAM superfamily; PF00271: Helicase conserved C-terminal domain; PF02518: Histidine kinase-, DNA gyrase B-, and HSP90-like ATPase; PF00535: Glycosyl transferase family 2; PF00155: Aminotransferase class I and II; PF00512: His Kinase A (phospho-acceptor) domain; PF00528: Binding-protein-dependent transport system inner membrane component; PF01381: Helix-turn-helix; PF00009: Elongation factor Tu GTP binding domain; PF00486: Transcriptional regulatory protein, C terminal; PF01926: 50S ribosome-binding GTPase; PF07992 : Pyridine nucleotide-disulphide oxidoreductase; PF07690: Major Facilitator Superfamily; PF00583: Acetyltransferase (GNAT) family; PF00664: ABC transporter transmembrane region; PF00534: Glycosyl transferases group 1; PF13419: Haloacid dehalogenase-like hydrolase; PF00440: Bacterial regulatory proteins, tetR family; PF01966: HD domain; PF00004: ATPase family associated with various cellular activities (AAA); PF00270: DEAD/DEAH box helicase; PF00132: Bacterial transferase hexapeptide (six repeats); PF00392: Bacterial regulatory proteins, gntR family; PF00126: Bacterial regulatory helix-turn-helix protein, lysR family; PF00672: HAMP domain; PF00293: NUDIX domain; PF03466: LysR substrate binding domain; PF00753: Metallo-beta-lactamase superfamily. A, osteoarthritis group; B, healthy control group; OA, osteoarthritis; *Q*, *P* value after adjusting for gender, age, and body mass index (BMI). #, mean ± standard deviation; ##, median. *, *P* < 0.05, statistical difference; **, *P* < 0.001, dramatically statistical difference; &, Q < 0.05, statistical difference.


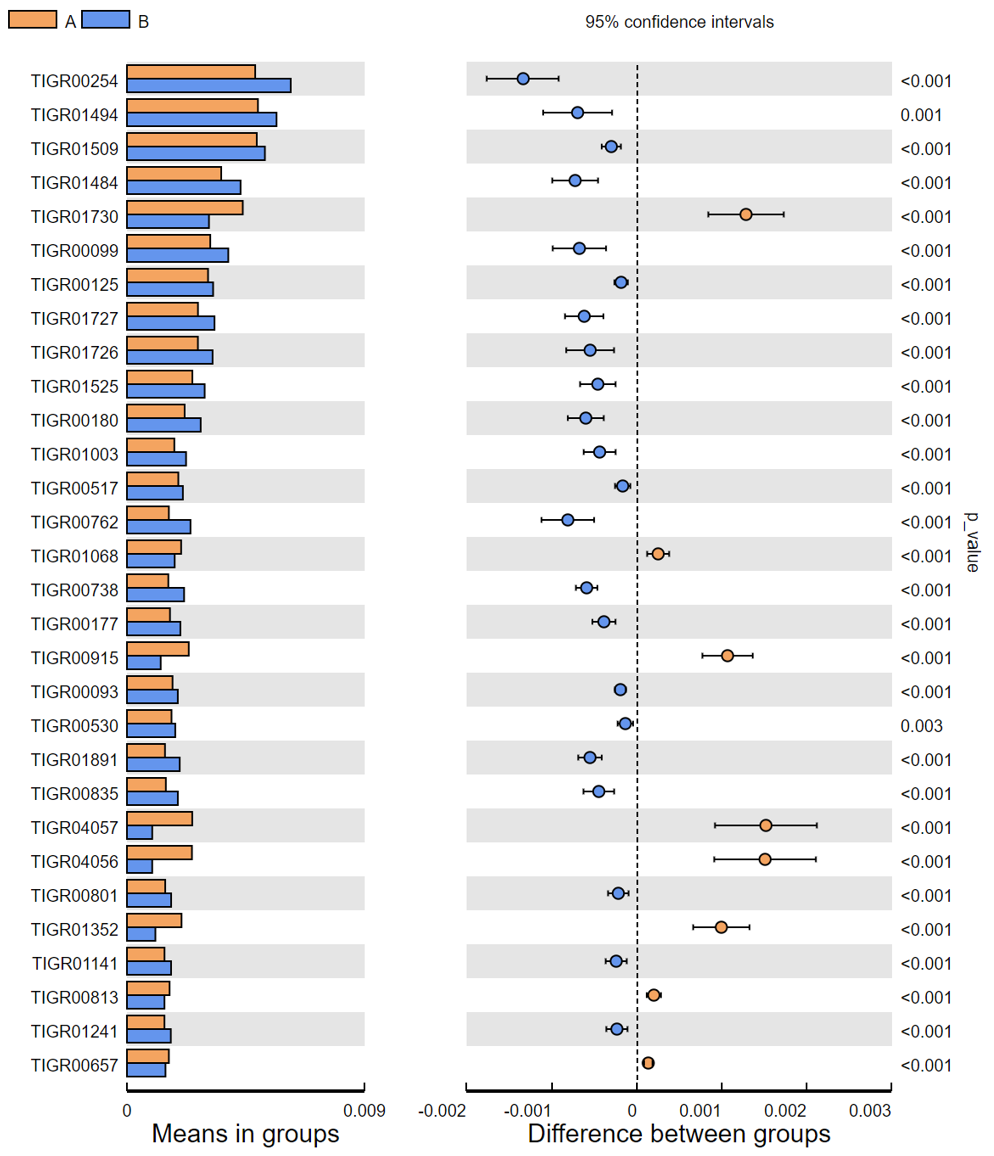


b

a

| Function | OA | Control | *P* | *Q* |
| --- | --- | --- | --- | --- |
| TIGR00254 | 131344±41287^#^ | 164173±27796^#^ | <0.001^**^ | 0.088 |
| TIGR01494 | 131050±25168^#^ | 148111±19682^#^ | 0.001^*^ | 0.407 |
| TIGR01509 | 130770^##^ | 144812^##^ | 0.078 | — |
| TIGR01484 | 95285±22620^#^ | 113310±15989^#^ | <0.001^**^ | **0.042**^&^ |
| TIGR01730 | 116350±33689^#^ | 82702±26179^#^ | <0.001^**^ | 0.114 |
| TIGR00099 | 83893±22107^#^ | 100758±17012^#^ | <0.001^**^ | 0.118 |
| TIGR00125 | 81661±11898^#^ | 86085±8451^#^ | 0.059 | — |
| TIGR01727 | 65237^##^ | 86345^##^ | 0.001^*^ | 0.074 |
| TIGR01726 | 63149^##^ | 81207^##^ | 0.008^*^ | **0.037**^&^ |
| TIGR01525 | 65533±13614^#^ | 76847±8816^#^ | <0.001^**^ | 0.357 |
| TIGR00180 | 577804±12632^#^ | 72879±10056^#^ | <0.001^**^ | 0.883 |
| TIGR01003 | 48491±17615^#^ | 59381±10966^#^ | 0.001^*^ | **0.042**^&^ |
| TIGR00517 | 51602±7977^#^ | 55620±4532^#^ | 0.007^*^ | **0.012**^&^ |
| TIGR00762 | 41835±18134^#^ | 62366±16194^#^ | <0.001^**^ | 0.821 |
| TIGR01068 | 54246±10248^#^ | 47385±3444^#^ | <0.001^**^ | 0.062 |
| TIGR00738 | 41984±10738^#^ | 56816±7022^#^ | <0.001^**^ | 0.258 |
| TIGR00177 | 40043^##^ | 54102^##^ | <0.001^**^ | 0.149 |
| TIGR00915 | 62387±22934^#^ | 34531±17429^#^ | <0.001^**^ | 0.071 |
| TIGR00093 | 45961±8023^#^ | 50890±5906^#^ | 0.002^*^ | **0.025**^&^ |
| TIGR00530 | 43949^##^ | 47647^##^ | 0.014^*^ | 0.224 |
| TIGR01891 | 37032^##^ | 51174^##^ | <0.001^**^ | 0.999 |
| TIGR00835 | 38568±8094^#^ | 49854±8801^#^ | <0.001^**^ | 0.608 |
| TIGR04057 | 60528^##^ | 22436^##^ | <0.001^**^ | 0.581 |
| TIGR04056 | 60367^##^ | 22431^##^ | <0.001^**^ | 0.558 |
| TIGR00801 | 34387^##^ | 42450^##^ | 0.003^*^ | 0.056 |
| TIGR01352 | 53915±24143^#^ | 28266±10364^#^ | <0.001^**^ | 0.482 |
| TIGR01141 | 37540±7347^#^ | 43620±6176^#^ | <0.001^**^ | 0.686 |
| TIGR00813 | 42018^##^ | 35095^##^ | 0.013^*^ | **0.003**^&^ |
| TIGR01241 | 37445±7524^#^ | 43370±5813^#^ | <0.001^**^ | 0.687 |
| TIGR00657 | 42063±5555^#^ | 38553±4467^#^ | 0.003^*^ | **0.012**^&^ |

TIGRFAM: Predicted function (Top 30) of gut microbiota based on TIGRFAM analysis. a, the histogram of relative abundance; b, the table of absolute abundance. TIGR00254: diguanylate cyclase (GGDEF) domain; TIGR01494: HAD ATPase, P-type, family IC; TIGR01509: HAD hydrolase, family IA, variant 3; TIGR01484: HAD hydrolase, family IIB; TIGR01730: efflux transporter, RND family, MFP subunit; TIGR00099: Cof-like hydrolase; TIGR00125: cytidyltransferase-like domain; TIGR01727: oligopeptide/dipeptide ABC transporter, ATP-binding protein, C-terminal domain; TIGR01726: amino ABC transporter, permease protein, 3-TM region, His/Glu/Gln/Arg/opine family; TIGR01525: heavy metal translocating P-type ATPase; TIGR00180: ParB/RepB/Spo0J family partition protein; TIGR01003: phosphocarrier, HPr family; TIGR00517: acyl carrier protein; TIGR00762: EDD domain protein, DegV family; TIGR01068: thioredoxin; TIGR00738: Rrf2 family protein; TIGR00177: molybdenum cofactor synthesis domain; TIGR00915: RND transporter, hydrophobe/amphiphile efflux-1 (HAE1) family; TIGR00093: pseudouridine synthase; TIGR00530: 1-acylglycerol-3-phosphate O-acyltransferases; TIGR01891: amidohydrolase; TIGR00835: amino acid carrier protein; TIGR04057: TonB-dependent outer membrane receptor, SusC/RagA subfamily, signature region; TIGR04056: TonB-linked outer membrane protein, SusC/RagA family; TIGR00801: uracil-xanthine permease; TIGR01352: TonB family C-terminal domain; TIGR01141: histidinol-phosphate transaminase; TIGR00813: transporter, solute:sodium symporter (SSS) family; TIGR01241: ATP-dependent metallopeptidase HflB; TIGR00657: aspartate kinase. A, osteoarthritis group; B, healthy control group; OA, osteoarthritis; *Q*, *P* value after adjusting for gender, age, and body mass index (BMI). #, mean ± standard deviation; ##, median. *, *P* < 0.05, statistical difference; **, *P* < 0.001, dramatically statistical difference; &, Q < 0.05, statistical difference.
